# piR‐RCC Suppresses Renal Cell Carcinoma Progression by Facilitating YBX‐1 Cytoplasm Localization

**DOI:** 10.1002/advs.202414398

**Published:** 2025-05-24

**Authors:** Ruyue Wang, Fan Li, Yudong Lin, Zeyi Lu, Wenqin Luo, Zhehao Xu, Ziwei Zhu, Yi Lu, Xudong Mao, Yang Li, Zhinian Shen, Haohua Lu, Yining Chen, Liqun Xia, Mingchao Wang, Lifeng Ding, Gonghui Li

**Affiliations:** ^1^ Department of Urology Sir Run Run Shaw Hospital Zhejiang University School of Medicine Hangzhou 310016 China

**Keywords:** nanoparticle, PIWI‐interacting RNAs, renal cell carcinoma, STAT3, YBX‐1

## Abstract

PIWI‐interacting RNAs (piRNAs), a novel category of small non‐coding RNAs, are widely expressed in eukaryotes and deregulated in several pathologies, including cancer. Little is known about their function and mechanism in renal cell carcinoma (RCC) progression. Herein, a down‐regulated piRNA in RCC, termed piR‐hsa‐28489 (designated as piR‐RCC), is identified to impede RCC progression both in vivo and in vitro. Mechanistically, piR‐RCC directly interacts with Y‐box binding protein 1 (YBX‐1), thus impeding p‐AKT‐mediated YBX‐1 phosphorylation and its subsequent nuclear translocation. Moreover, YBX‐1 coordinates the transcription of ETS homologous factor (EHF) as a repressor factor. Consequently, piR‐RCC enhances EHF expression, leading to the inhibition of RCC proliferation and metastasis. Based on these, a biomimetic nanoparticle platform is constructed to achieve RCC‐specific targeted delivery of piR‐RCC. The nanoparticles are fabricated using a cell membrane coating derived from cancer cells and used to encapsulate and deliver piR‐RCC plasmids to renal orthotopic implantation in mice, hindering RCC progression. This study illustrates piR‐RCC/YBX‐1/EHF signaling axis in RCC, offering a promising therapeutic avenue for RCC.

## Graphical abstract

PIWI‐interacting RNAs (piRNAs), a novel category of small non‐coding RNAs, have been implicated in the development of various diseases. This study explores the tumor‐suppressive mechanism of a downregulated piRNA (designated piR‐RCC) in renal cell carcinoma (RCC), and provides a delivery strategy targeting RCC tumor by constructing a cell membrane‐camouflaged nanoparticles system.

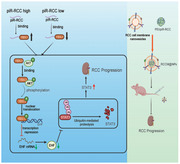

## 1 Introduction

Renal cell carcinoma (RCC) is the most common malignant tumor of the renal parenchyma, accounting for ≈90% of renal malignancies and 3–5% of all malignant tumors.^[^ ^1^ ^]^ RCC typically exhibits a gradual progression, with ≈30% of patients with localized disease ultimately developing metastasis.^[^ ^1^, ^2^, ^3^ ^]^ The incidence of RCC is on the rise globally, ranking sixth among malignant tumors in men and ninth in women.^[^ ^4^ ^]^ However, the pathogenesis of RCC is highly complex, continually posing challenges to the treatment of this disease.

Piwi‐interacting RNA (piRNA) is a novel class of small non‐coding RNA molecules, ≈24–31 nucleotides in length. Initially prevalent in germ cells, piRNAs induce silencing of transposable elements in animal germ cells at both transcriptional and post‐transcriptional levels to maintain genomic integrity, as well as regulate protein‐coding genes in germ cells.^[^ ^5^ ^]^ Recent observations have extended the functional purview of piRNAs beyond the mammalian germline, with numerous studies delineating their regulatory impact across diverse tumor types.^[^ ^5^, ^6^ ^]^ However, despite these advances, research concerning piRNAs in tumorigenesis remains limited, leaving their biological functions and mechanisms within tumors still enigmatic to us.

Y‐box binding protein 1 (YBX‐1) is a multifunctional protein involved in many biological functions, of which functional diversity stems from interaction with a broad range of DNA and RNA molecules. Moreover, its function is also intricately linked to its subcellular localization. YBX‐1 predominantly localizes in the cytoplasm, where it binds to mRNA, regulating stability, translation, and other processes.^[^ ^7^, ^8^, ^9^, ^10^, ^11^, ^12^, ^13^ ^]^ Nuclear‐located YBX‐1 functions as a transcription factor or RNA splicing factor to participate in gene regulation.^[^ ^14^, ^15^, ^16^, ^17^ ^]^ Many studies have confirmed that YBX‐1 is involved in various tumorigenic events, including tumorigenesis, proliferation, metastasis, and drug resistance.^[^ ^18^, ^19^, ^20^, ^21^, ^22^, ^23^, ^24^ ^]^ However, the mechanism of YBX‐1 nuclear localization in RCC progression remains to be elucidated. Therefore, this study also focuses on the regulation of YBX‐1 nuclear localization by piR‐RCC and its impact on the transcription of downstream genes.

In recent years, biomimetic nanomedicines based on cell membrane cloaking technology have shown tremendous potential in biomedical applications, particularly in targeted drug delivery. Cell membrane coating technology is a top‐down biomimetic approach that utilizes natural cell membranes as nanocarriers to facilitate the delivery of core materials.^[^ ^25^, ^26^ ^]^ These coated nanoparticles are endowed with the inherent functions of the parent cells. The cancer cell membranes, which express “self‐markers ” and “self‐recognition molecules” can be wrapped around the surface of nanoparticles, granting them the natural ability to target homotypic tumor cells.^[^ ^27^, ^28^ ^]^ Compared to uncoated nanoparticles, cancer cell membrane‐coated nanoparticles can significantly enhance the stability of nanoparticles under physiological conditions and increase their accumulation in tumors through the homing effect.^[^ ^29^ ^]^

In this study, we demonstrated that piR‐hsa‐28489, which we renamed as piR‐RCC, is downregulated in RCC samples. Reduced expression of piR‐RCC is associated with poor prognosis in RCC patients. Furthermore, down‐regulated piR‐RCC promotes proliferation and metastasis both in vitro and in vivo. Mechanistically, piR‐RCC directly binds to YBX‐1, thereby interfering with the interaction between YBX‐1 and p‐AKT, which affects the phosphorylation level of YBX‐1, leading to the inhibition of YBX‐1 nuclear translocation. Nuclear‐localized YBX‐1 acts as a transcriptional repressor on ETS homologous factor (EHF). Ultimately, this promotes the transcription of EHF regulated by YBX‐1, affecting its downstream pathways and mediating the inhibition of RCC progression. Based on these, we constructed a cell membrane‐camouflaged nanoparticles system to encapsulate and deliver piR‐RCC plasmids to renal orthotopic implantation in mice, impeding RCC progression. Overall, our study offers a novel and promising therapeutic option for RCC.

## 2 Results

### 2.1 Identification of RCC‐Related piRNAs

**Figure 1:**
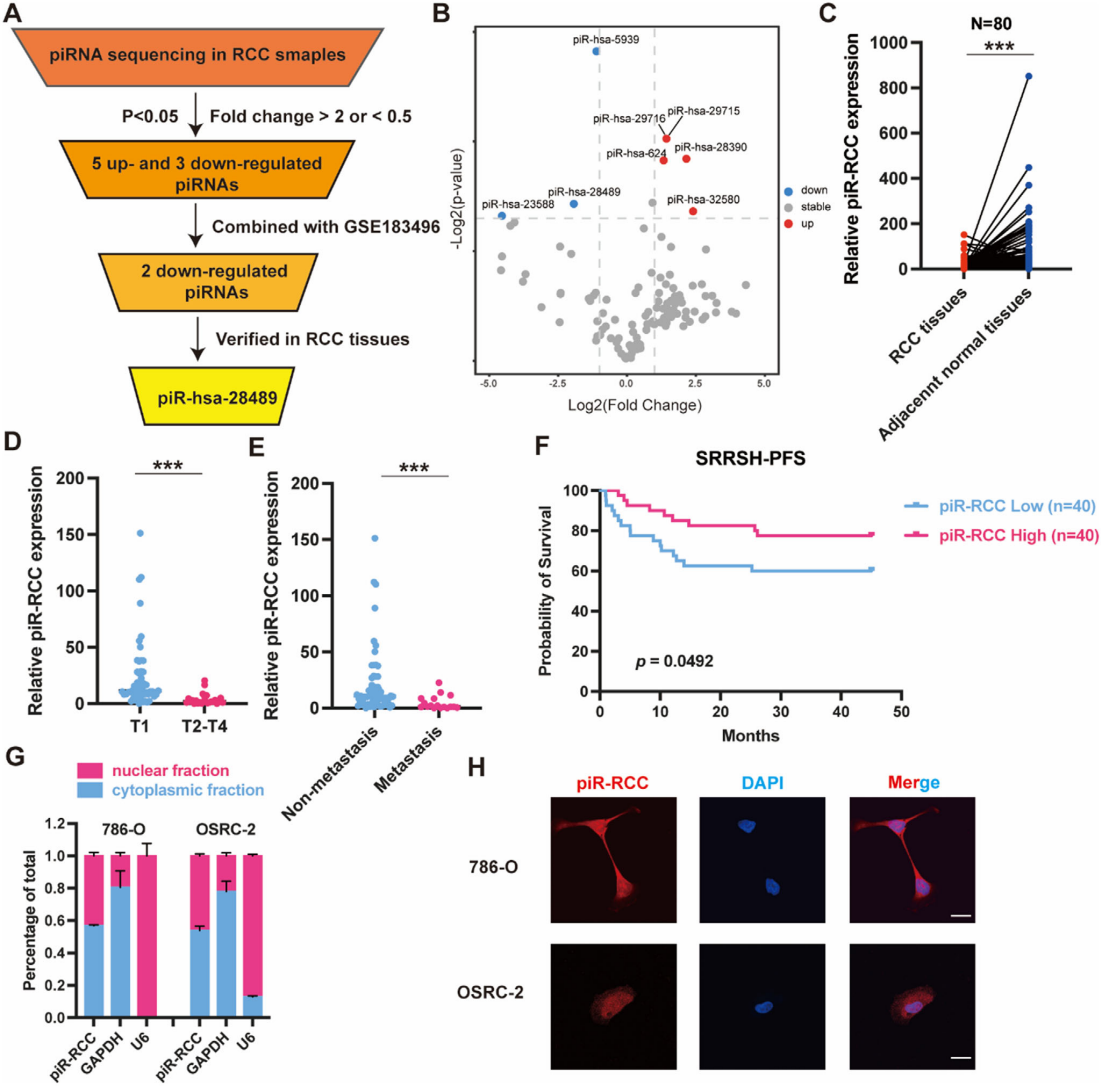
piR‐RCC is down‐regulated in RCC and associated with prognosis. A) Flowchart illustrates the selection processes of piR‐RCC based on RNA‐seq data. B) Volcano of piRNAs expression between RCC tissues and adjacent normal tissues. C) RT‐qPCR analysis of piR‐RCC in in RCC tissues and matched normal tissues from SRRSH cohort. D,E) piR‐RCC expression levels between T1 stage and T2‐T4 stage (D), non‐metastasis and metastasis groups (E) from SRRSH cohort measured by RT‐qPCR. F) Kaplan‒Meier survival curves of progression‐free‐survival in low and high piR‐RCC expression groups (n = 80). G) RT‐qPCR analysis of piR‐RCC in the nuclear/cytoplasmic fraction. GAPDH was used as controls which was mainly located in cytoplasm, while U6 was mainly located in nucleus. H) Fluorescence in situ hybridization assay showed the subcellular localization of piR‐RCC. Nuclei were stained with DAPI. Scale bar, 20 µm. Data are presented as mean ± SD, paired *t*‐test for (C), two‐side unpaired t test for (D,E); logrank test for (F). ^***^ *p* < 0.001.

To investigate the role of piRNAs in regulating the progression of renal cell carcinoma, we initially detected the global expression of piRNAs in 3 paired RCC tissues by piRNA sequencing (**Figure** **1**A,B). Subsequently, among the 133 piRNAs analyzed, we identified a total of 5 up‐regulated and 3 down‐regulated piRNAs using the criteria: *p* < 0.05 and | Log_2_(Fold change) | > 1. To narrow down the scope, we integrated these findings with our prior piRNA sequencing data (GSE183496), leading to the selection of two down‐regulated piRNAs (piR‐hsa‐28489 and piR‐hsa‐23588). Finally, we validated the expression of these two piRNAs in 26 paired RCC tissues. The results indicated that both piR‐hsa‐28489 and piR‐hsa‐23588 were down‐regulated in RCC tissues compared to normal tissues, with piR‐hsa‐28489 demonstrating a more pronounced difference (Figure S1A, Supporting Information). Consequently, piR‐hsa‐28489 was selected for further exploration. Considering piR‐hsa‐28489 was down‐regulated in RCC tissues and potentially involved in RCC progression, we, thus, named this uncharacterized piRNA as piR‐RCC. Subsequently, we evaluated the expression of piR‐RCC in a larger cohort containing 80 paired RCC tissues. Our findings demonstrated that piR‐RCC was down‐regulated in RCC tissues compared to normal tissues (Figure 1C). By analyzing the expression of piR‐RCC in conjunction with clinical characteristics of RCC patients, we found that piR‐RCC was more down‐regulated in RCC patients with T2‐T4 stage compared to those with T1 stage (Figure 1D). Moreover, the expression of piR‐RCC was diminished in metastatic RCC patients (Figure 1E). Utilizing the median value, the 80 RCC samples were divided into two groups based on piR‐RCC expression (high and low expression groups). Survival analysis showed that RCC patients with high piR‐RCC expression exhibited a promising prognosis (Figure 1F). Finally, cytoplasmic and nuclear fractionation as well as fluorescence in situ hybridization (FISH) assays showed the distribution of piR‐RCC in both the nucleus and cytoplasm of RCC cells (Figure 1G,H). Altogether, these data show the down‐regulation of piR‐RCC in RCC tissues and its negative correlation with malignant features.

### 2.2 piR‐RCC Restrains RCC Proliferation

**Figure 2:**
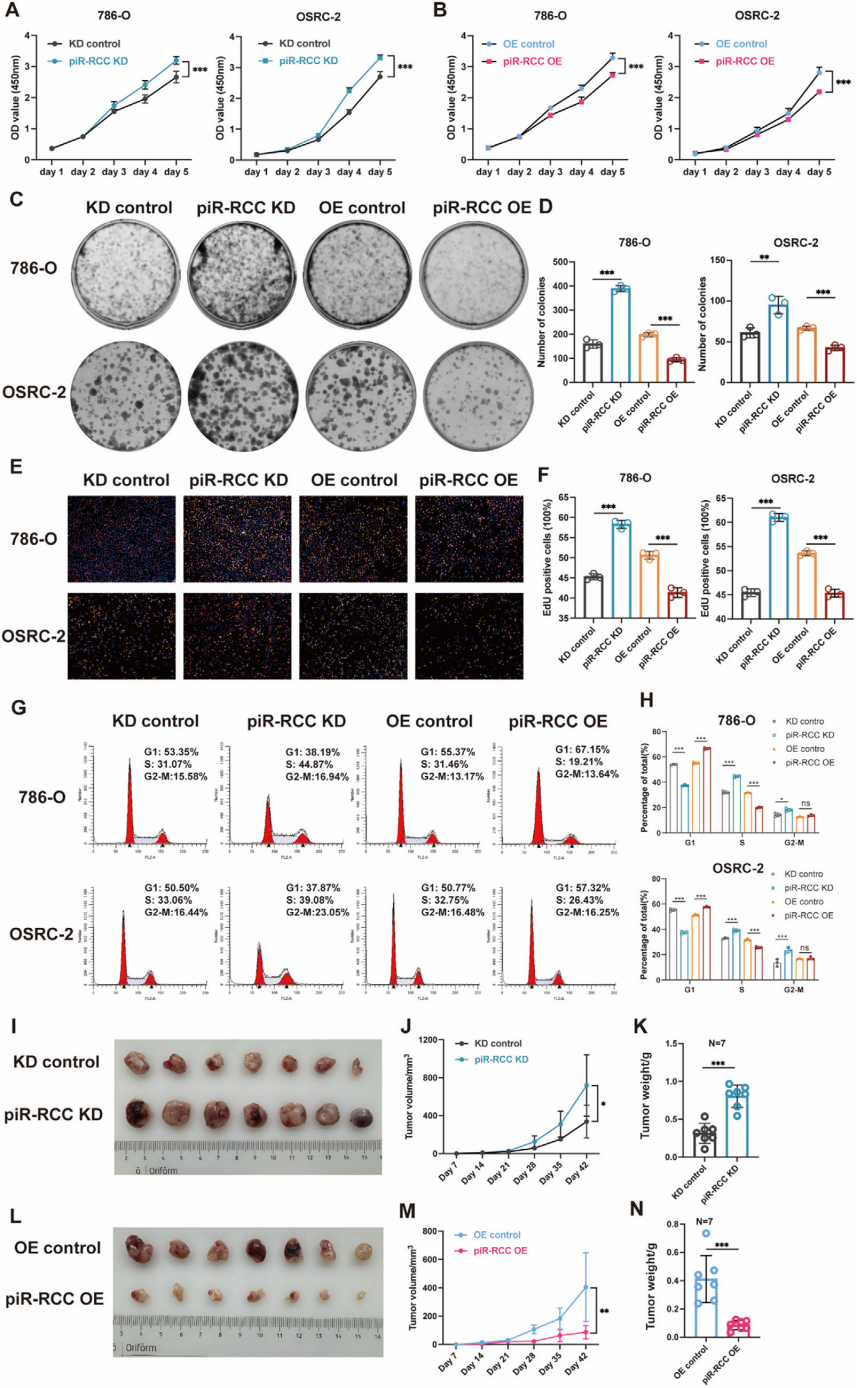
piR‐RCC restrains RCC proliferation in vitro and in vivo. A,B) CCK‐8 assays were performed to determine the proliferation ability for piR‐RCC knockdown (A) or overexpressing (B) in 786‐O and OSRC‐2 cells. C,D) Representative images of colony‐formation assay and its quantification data indicated 786‐O and OSRC‐2 cells. E,F) Representative images of EdU assay and its quantification data in indicated 786‐O and OSRC‐2 cells. Scale bar, 50µm. G,H) Flow cytometric analysis of cell cycle in piR‐RCC knockdown and overexpression cells. I–K) Images (I), volumes (J), and weights (K) of indicated cell–derived xenograft tumors in piR‐RCC knockdown and control groups (n = 7). L–N) Images (L), volumes (M), and weights (N) of indicated cell–derived xenograft tumors in piR‐RCC overexpression and control groups (n = 7). Data are presented as mean ± SD, two‐tailed unpaired *t*‐test for (A, B, D, F, H, J, K, M, N). ^*^ *p* < 0.05, ^**^ *p* < 0.01, ^***^ *p* < 0.001; ns, not significant.

To explore the role of piR‐RCC in RCC progression, we utilized piR‐RCC inhibitors to disrupt its function and piR‐RCC mimics to upregulate its expression. The overexpression efficiency of piR‐RCC mimics was verified (Figure S2A, Supporting Information). Subsequently, cell counting kit‐8 (CCK‐8), colony formation, and 5‐ethynyl‐20‐deoxyuridine (EdU) assays were applied to detect the role of piR‐RCC on the proliferation of RCC cells. The results indicated that piR‐RCC knockdown significantly increased the proliferation ability of RCC cells, while piR‐RCC overexpression suppressed their proliferation (**Figure** **2**A–F). Furthermore, flow cytometry assays indicated that altered piR‐RCC expression did not change the apoptotic ratio of RCC cells (Figure S2B,C, Supporting Information), while cell cycle analysis demonstrated that piR‐RCC induced arrest of RCC cells at the G1 phase (Figure 2G,H). Furthermore, xenograft animal model demonstrated that piR‐RCC knockdown promoted RCC cell proliferation in vivo (Figure 2I‐K), while piR‐RCC overexpression restrained RCC cell proliferation in vivo (Figure 2L‐N). In total, these data show that piR‐RCC suppresses RCC proliferation both in vitro and in vivo.

### 2.3 piR‐RCC Suppresses RCC Metastasis

**Figure 3:**
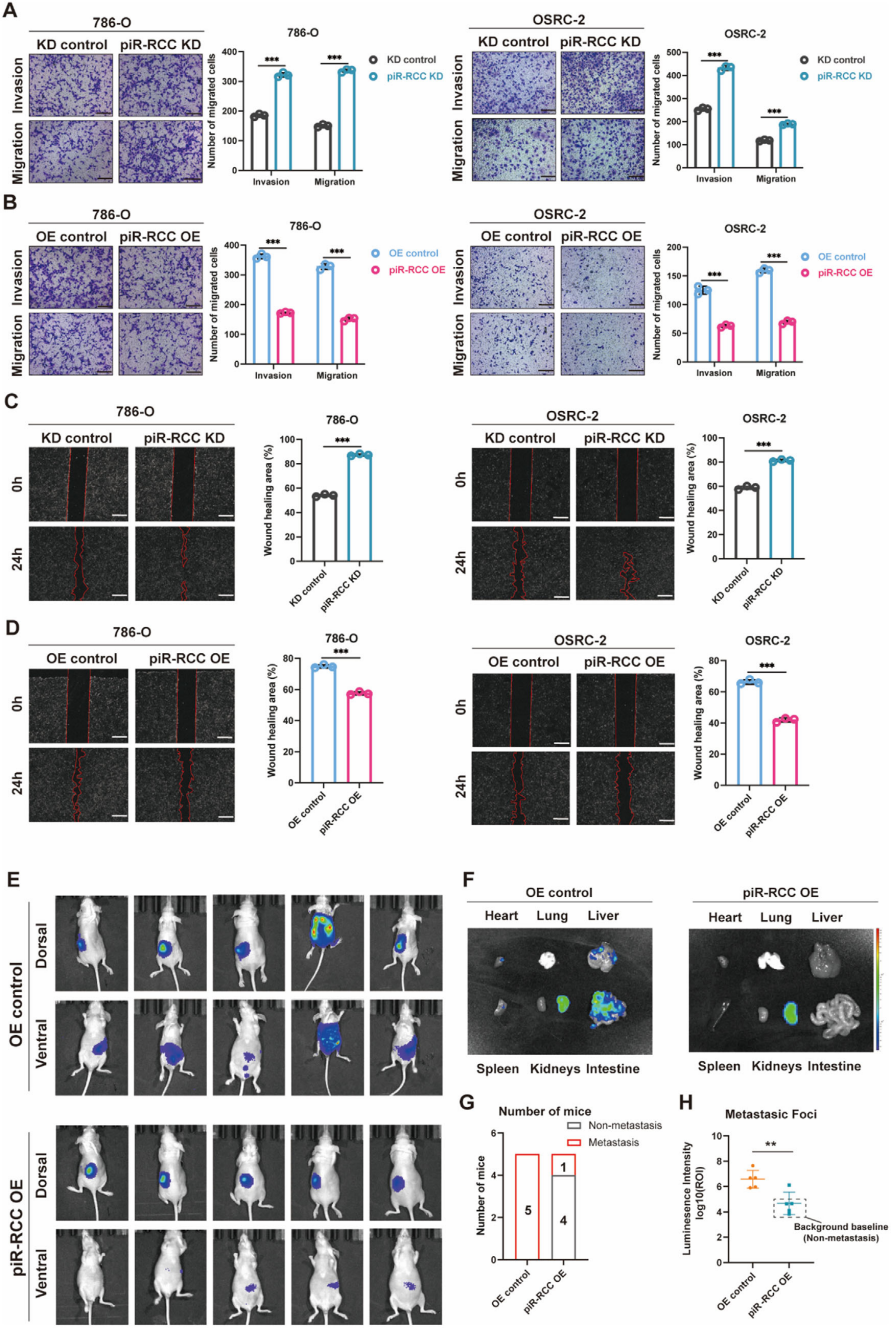
piR‐RCC suppresses RCC metastasis in vitro and in vivo. A,B) Effects of piR‐RCC on the capability of invasion and migration of RCC cells was evaluated by transwell assay. Scale bar, 100 µm. C,D) Effect of piR‐RCC knockdown or overexpression on the migration ability of RCC cells was evaluated by wound healing assay. Scale bar, 100 µm. E) Bioluminescent images showing primary foci and metastases in mice following luciferase‐labeled ACHN cells injection under the renal capsule of left kidney (n = 5). F) Representative bioluminescent images of *ex vivo* organs and metastatic lesions. G) Quantification of the number of mice with metastases described in (E). H) Bioluminescent signal intensities (photons/s/cm^2^/sr) of metastatic foci were quantified (for mice without tumor metastasis, the background baseline bioluminescence of their organs was used to substitute for metastatic foci intensity in statistical analyses). Data are presented as mean ± SD, two‐tailed unpaired *t*‐test for (A to F) ^*^ *p* < 0.05, ^**^ *p* < 0.01, ^***^ *p* < 0.001; ns, not significant.

Subsequently, we evaluated the role of piR‐RCC on the metastasis ability of RCC cells. Transwell and wound healing assays revealed that piR‐RCC knockdown significantly enhanced the metastasis of RCC cells, whereas piR‐RCC overexpression restrained their metastatic ability (**Figure** **3**A–D). Furthermore, to assess piR‐RCC's role in tumor metastasis in vivo, we orthotopically injected luciferase‐labeled ACHN RCC cells in nude mice. The results demonstrated that in the piR‐RCC overexpression group, tumor metastasis was inhibited, highlighting the direct effect of piR‐RCC on RCC metastasis (Figure 3E–H). Overall, these data strongly suggest that piR‐RCC acts as a suppressor of RCC metastasis.

### 2.4 piR‐RCC Directly Interacts with YBX‐1 Proteins in RCC Cells

**Figure 4:**
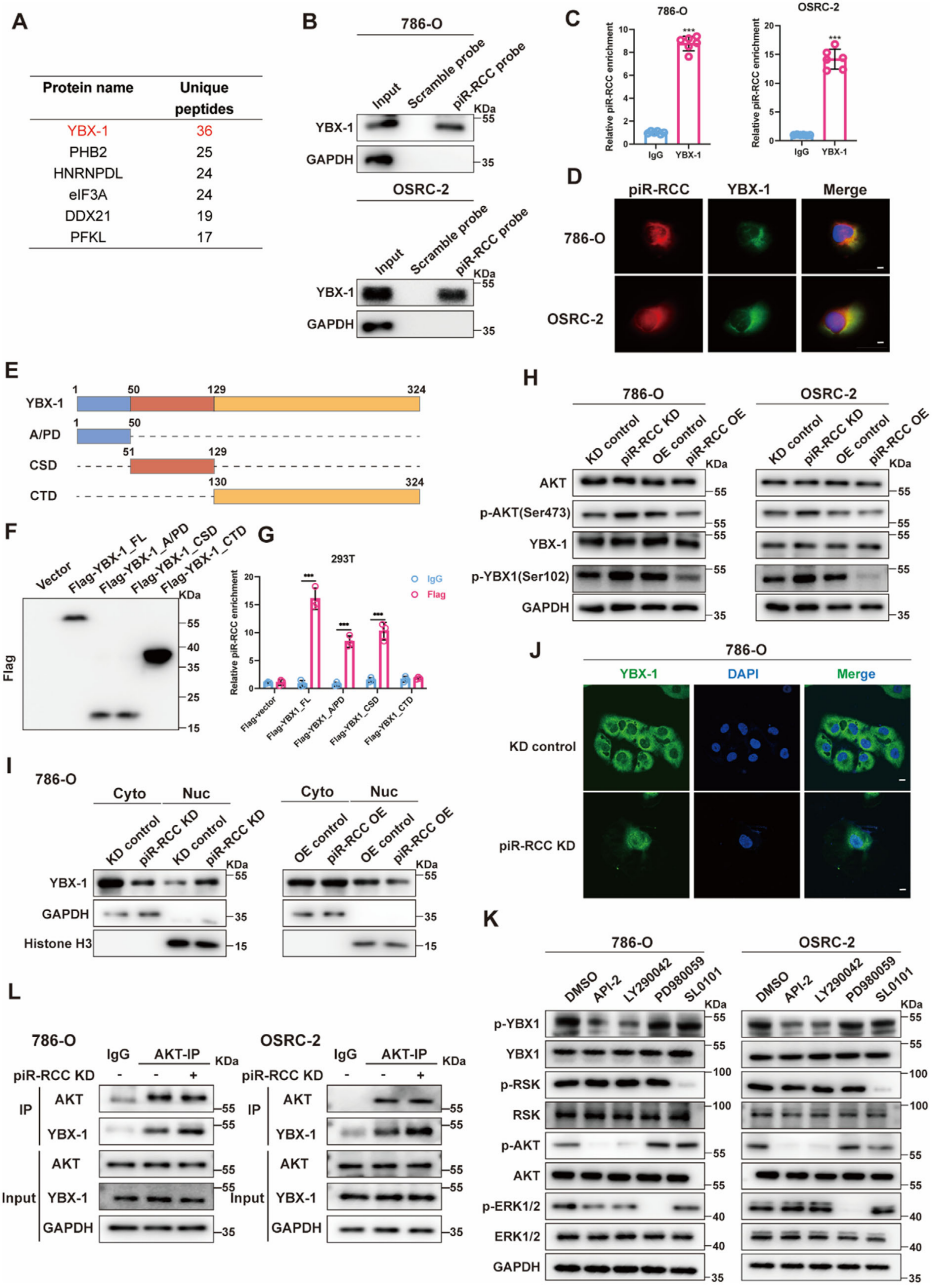
piR‐RCC interacts with YBX‐1 in RCC cells and suppresses its nuclear translocation. A) Mass spectrometry analysis of proteins associated with piR‐RCC. B) Immunoblots of YBX‐1 enriched by biotinylated scramble or piR‐RCC probes in 786‐O and OSRC‐2 cells. C) RIP assays verified the binding between YBX‐1 and piR‐RCC. D) FISH and IF assays were used to detect the colocalization of piR‐RCC and YBX‐1. Nuclei were labeled with DAPI (blue). Scale bar, 10µm. E) Schematic depicting the structure and sequence of YBX‐1. Blue box, the A/PD rich domain; red box, an evolutionarily conserved cold‐shock domain (CSD); yellow box, the C‐terminal domain (CTD). F) Western blotting analysis was used to verify the expression efficiencies of full‐length and truncated YBX‐1. G) RIP assays confirmed the interaction between YBX‐1 truncations and piR‐RCC. H) WB analysis of p‐YBX‐1(Ser102), total YBX1, p‐AKT (Ser473), total AKT in piR‐RCC knockdown or overexpression cells. I) WB analysis of YBX‐1 in cytoplasmic and nuclear fractions of piR‐RCC knockdown or overexpression 786‐O cells. Histone H3 was used as a nuclear control. GAPDH was used as a cytoplasmic control. J) IF assays showed the subcellular localization of YBX‐1 protein in piR‐RCC knockdown or control cells. Scale bar, 10µm. K) Western blotting of the p‐YBX‐1, p‐RSK, p‐AKT, and p‐ERK1/2 and their total protein levels in 786‐O and OSRC‐2 cells treated with API‐2, LY294002, PD098059 or SL0101. L) Co‐IP assay of AKT and YBX‐1 in 786‐O and OSRC‐2 cells with piR‐RCC knockdown. Data presented as mean ± SD, two‐tailed unpaired *t*‐test was used for (C, G). ^***^ *p* < 0.001.

To uncover the molecular mechanism underlying piR‐RCC's regulation of RCC progression, we initially evaluated the cis‐regulation ability of piR‐RCC on nearby extrachromosomal genes. Therefore, a qRT‐PCR assay was employed to investigate whether piR‐RCC regulates the expression of these seven genes (BTN1A1, BTN2A1, BTN3A3, ZNF322, HMGN4, ABT1, and HCG11) within ≈0.3 mega‐bases centered on the piR‐RCC gene locus. The results showed that piR‐RCC has minimal influence on these genes' expression (Figure S3A, Supporting Information), suggesting that piR‐RCC does not function in this way. Next, RNA pulldown was conducted to explore the piR‐RCC‐binding proteins using piR‐RCC probes and scramble probes. RNA pulldown combined with mass spectrometry was conducted (Table S7, Supporting Information). We found that piR‐RCC could bind to YBX‐1 proteins, which had the highest unique peptide numbers in piR‐RCC group (**Figure** **4**A). RNA pulldown followed by western blot confirmed the direct binding of piR‐RCC and YBX‐1 (Figure 4B). YBX‐1‐RNA immunoprecipitation (RIP) assay also indicated the interaction of YBX‐1 and piR‐RCC (Figure 4C). Furthermore, FISH and Immunofluorescence (IF) assays also validated their interaction (Figure 4D). Subsequently, we investigated the specific regions of YBX‐1 binding to piR‐RCC. YBX‐1 is composed of an N‐terminal alanine/proline‐rich domain (A/PD), a cold shock domain (CSD), as well as a C‐terminal domain (CTD) (Figure 4E). Thus, we designed the YBX‐1 full‐length and truncation plasmids with a 3x Flag tag. The successful construction of these plasmids was verified by western blot (Figure 4F). The RIP assays showed that piR‐RCC was enriched by the A/PD and CSD domains of YBX‐1, while it could not be enriched by the CTD domain of YBX‐1 (Figure 4G). Furthermore, RNA pulldown assays using piR‐RCC probes revealed that piR‐RCC specifically interacted with the full‐length, A/PD, and CSD domains of YBX‐1 (Figure S3B, Supporting Information). Then HDOCK was used to predict the molecular docking between piR‐RCC and YBX‐1 protein (Figure S3C, Supporting Information). Altogether, these data reveal that piR‐RCC directly interacts with YBX‐1 proteins.

Furthermore, we tend to study the effect of interaction between piR‐RCC and YBX‐1. Despite previous indications of YBX‐1′s involvement in post‐transcriptional control of various mRNA splicing, altering the expression of YBX‐1 did not affect piR‐RCC expression (Figure S3D,E, Supporting Information). Then, we explored the effect of piR‐RCC on YBX‐1 expression, and the results showed that piR‐RCC knockdown elevated the phosphorylation level of YBX‐1 at Ser102, while piR‐RCC overexpression reduced this phosphorylation level, with no discernible impact on its protein level (Figure 4H). In addition, we verified that piR‐RCC had minimal effect on YBX‐1 mRNA expression (Figure S3F, Supporting Information). Previous studies revealed that phosphorylation of YBX‐1 at Ser102 is essential for its nuclear translocation in cancer cells.^[^ ^14^, ^30^, ^31^, ^32^, ^33^, ^34^, ^35^ ^]^ We, thus, explored the role of piR‐RCC in YBX‐1 subcellular distribution. Cytoplasmic and nuclear protein fractionation assays demonstrated that piR‐RCC decreased the nuclear localization of YBX‐1 (Figure 4I; Figure S3G, Supporting Information). Moreover, IF assays revealed that piR‐RCC knockdown promoted YBX‐1 nuclear localization (Figure 4J; Figure S3H, Supporting Information). These data show that piR‐RCC suppresses YBX‐1 nuclear translocation via decreasing phosphorylation level of YBX‐1 at Ser102.

P‐RSK, p‐AKT, and p‐ERK1/2 have been reported to phosphorylate YBX‐1 at Ser102 (S102). To discern the specific pathway involved in piR‐RCC‐induced YBX‐1 phosphorylation in RCC, RSK inhibitor (SL0101), PI_3_K‐AKT pathway inhibitors (API‐2 and LY290042), and p‐ERK1/2 inhibitor (PD980059) were employed to treat RCC cells.^[^ ^23^, ^36^, ^37^, ^38^, ^39^ ^]^ Western blot assay revealed that API‐2 and LY290042, two PI_3_K‐AKT pathway inhibitors, notably reduced the p‐YBX‐1 (S102) level in RCC cells, while RSK and ERK1/2 inhibitors exhibited negligible impact on its expression (Figure 4K). Additionally, we detected the functional effect of piR‐RCC on p‐AKT level. We found that p‐AKT (Ser473) level was increased upon piR‐RCC knockdown, whereas p‐AKT (Ser473) level was decreased upon piR‐RCC overexpression (Figure 4H), supporting the role of piR‐RCC in regulating the p‐AKT‐mediated YBX‐1 phosphorylation. Furthermore, Co‐Immunoprecipitation (Co‐IP) assays using AKT antibodies confirmed a direct interaction between YBX‐1 and AKT in RCC cells (Figure S4A, Supporting Information). Notably, piR‐RCC knockdown enhanced the YBX‐1/p‐AKT interaction in RCC cells (Figure 4L). To investigate whether piR‐RCC modulates YBX‐1 Ser102 phosphorylation through AKT phosphorylation, we treated cells with API‐2, a PI_3_K‐AKT pathway inhibitor. Western blot analysis revealed that piR‐RCC knockdown had minimal impact on YBX‐1 Ser102 phosphorylation under API‐2 treatment (Figure S4B, Supporting Information), suggesting that piR‐RCC regulates YBX‐1 Ser102 phosphorylation through the AKT pathway. Furthermore, cytoplasmic and nuclear protein fractionation assays demonstrated that piR‐RCC had little effect on YBX‐1 nuclear localization under API‐2 treatment (Figure S4C, Supporting Information), indicating that piR‐RCC regulates YBX‐1 nuclear localization via the AKT pathway. To investigate the role of YBX‐1 Ser102 phosphorylation in YBX‐1 nuclear localization, we generated a YBX‐1 S102 mutant (YBX‐1 S102A). Our results demonstrated that SC79, an AKT activator, significantly enhanced YBX‐1 nuclear import. However, SC79 had little effect on the nuclear localization of the YBX‐1 S102A mutant (Figure S4D, Supporting Information), indicating that Ser102 is a crucial site for AKT‐dependent YBX‐1 phosphorylation and subsequent nuclear translocation. Collectively, these data demonstrate that piR‐RCC suppresses p‐AKT‐dependent YBX‐1 Ser102 phosphorylation and subsequently reduces YBX‐1 nuclear translocation.

### 2.5 piR‐RCC and YBX‐1 Co‐Regulate EHF Transcription

**Figure 5:**
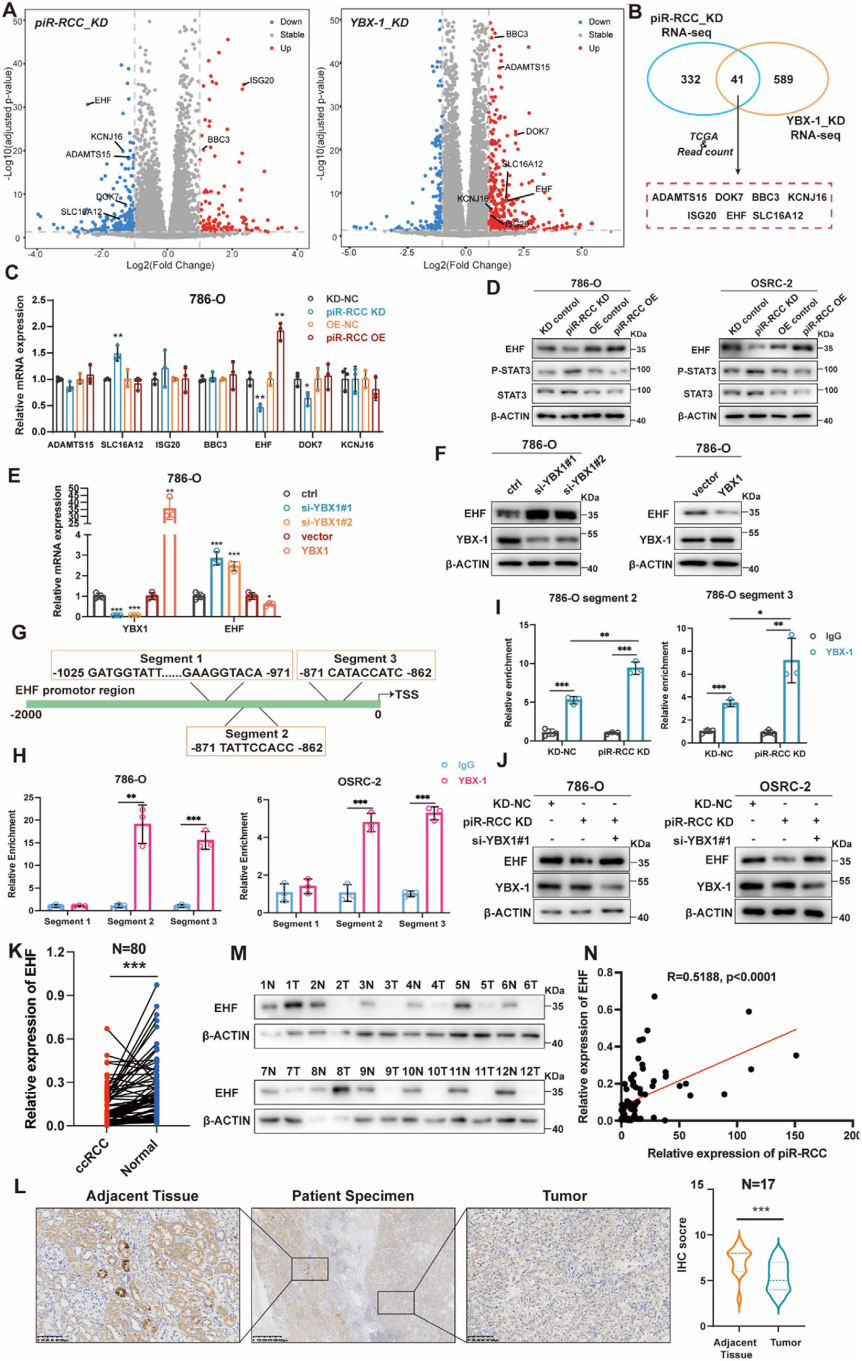
piR‐RCC and YBX‐1 co‐regulate EHF transcription. A) Volcano plots respectively showing differentially expressed RNAs in 786‐O upon piR‐RCC knockdown (left) and in ACHN upon YBX‐1 knockdown (right). B) Workflow for screening downstream targets of YBX‐1 and piR‐RCC. C) qRT‐PCR was conducted to detect the mRNA expression of candidate genes as indicated. D) Effects of piR‐RCC on the protein level of EHF and downstream STAT3 detected by western blotting. E,F) qRT‐PCR and western blotting analysis of EHF expression in YBX‐1 knockdown or overexpressing 786‐O cells. G) The putative YBX‐1 binding sites in EHF promoter predicted using the JASPAR database. H) ChIP assay confirmed the interaction between YBX‐1 and EHF promoter segments. I) YBX‐1‐ChIP‐qPCR assays demonstrated that piR‐RCC knockdown increased the binding capacity between YBX‐1 and segment 2/3. J) Western blotting showing the protein level of EHF in RCC cells transfected with indicated plasmids and siRNAs. K) qRT‐PCR analysis displaying the mRNA levels of EHF in paired RCC tumor and normal tissues from SRRSH RCC cohort. L) Representative IHC staining images for EHF protein in the SRRSH RCC cohort are presented. IHC scores are calculated and analyzed. M) Western blot assay displaying the protein levels of EHF in paired RCC tumor and normal tissues from SRRSH RCC cohort. N) Spearman's correlations between piR‐RCC and EHF in RCC tissues. ^*^ *p* < 0.05, ^**^ *p* < 0.01, ^***^ *p* < 0.001; ns, not significant. Data presented as mean ± SD, two‐tailed unpaired *t*‐test was used for (C, E, I, H), paired *t*‐test for (K), Wilcoxon test for (L), Spearman's correlations for (N). ^*^ *p* < 0.05, ^**^ *p* < 0.01, ^***^ *p* < 0.001.

To explore the downstream genes regulated by piR‐RCC, RNA‐seq was performed using 786‐O cells with piR‐RCC knockdown or control. A total of 373 genes were found to be dysregulated upon piR‐RCC knockdown (**Figure** **5**A). To refine our focus and screen potential targets, we conducted transcriptome profiling in ACHN cells with YBX‐1 knockdown (Figure 5A). Subsequent integrative analysis of differentially expressed genes from both piR‐RCC and YBX‐1 knockdown RNA‐seq datasets identified 41 common differentially expressed genes. Further filtration of these candidates based on read count in RNA‐seq and differential expression status in the Cancer Genome Atlas (TCGA) renal cell carcinoma cohort yielded 7 potential downstream targets of piR‐RCC (Figure 5B). Validation of 7 selected candidates by qRT‐PCR in RCC cells with piR‐RCC knockdown or overexpression demonstrated that piR‐RCC predominantly upregulates EHF expression, while exerting minimal regulatory effects on other candidates (Figure 5C; Figure S5A, Supporting Information). The western blot assay further validated these findings (Figure 5D). Furthermore, qRT‐PCR and western blot assays indicated that YBX‐1 negatively regulated EHF expression (Figure 5E,F; Figure S5B,C, Supporting Information). We further explored how YBX‐1 regulates EHF expression in RCC cells. EHF mRNA stability assays revealed that YBX‐1 had no discernible impact on EHF mRNA stability (Figure S5D, Supporting Information), suggesting that YBX‐1 did not regulate EHF mRNA expression at the post‐transcriptional level. Considering that once localized in the nucleus, YBX‐1 acts as a transcriptional factor regulating the expression of various tumor‐associated genes.^[^ ^40^ ^]^ We, thus, hypothesized that YBX‐1 regulates EHF expression at the transcriptional level. By utilizing JASPAR database, three potential YBX‐1 binding sites in the promoter region of EHF were predicted (Figure 5G). YBX‐1‐Chromatin Immunoprecipitation (ChIP)‐qPCR assays showed that YBX‐1 directly binds to EHF promoter region via the segment 2 and segment 3 (Figure 5H). Furthermore, YBX‐1‐ChIP‐qPCR assays demonstrated that piR‐RCC knockdown enhanced the binding between YBX‐1 and segment 2/3 (Figure 5I), while exerting minimal effect on segment 1 (Figure S5E, Supporting Information), consistent with the observation that piR‐RCC knockdown facilitated the nuclear localization of YBX‐1. Moreover, rescue assays indicated that the inhibitory effect of piR‐RCC knockdown on EHF expression could be reversed by YBX‐1 knockdown in RCC cells (Figure 5J). Finally, we analyzed the expression of EHF in TCGA‐KIRC database and our 80 RCC samples. The results indicated that EHF was down‐regulated in RCC tissues compared to adjacent normal tissues at the mRNA level (Figure S5F, Supporting Information; Figure 5K). Western blot and immunohistochemistry (IHC) assays also further confirmed the downregulated EHF protein level in RCC samples (Figure 5L,M). Meanwhile, EHF was down‐regulated in RCC patients with advanced stage or metastasis (Figure S5G,H, Supporting Information). Correlation analysis of piR‐RCC and EHF in RCC tissues demonstrated the positive correlation between piR‐RCC and EHF (Figure 5N). Taken together, these data show that piR‐RCC orchestrates EHF transcription in a YBX‐1‐dependent manner.

### 2.6 piR‐RCC Restrains RCC Progression via Regulating EHF/STAT3 Axis

Subsequently, we investigated the biological role of EHF in RCC progression. By utilizing CCK‐8 and colony formation assays, we found that EHF acted as a tumor‐suppressor, restraining RCC cell proliferation (Figure S6A–C, Supporting Information). Moreover, cell cycle analysis unveiled that EHF induced the arrest of RCC cells at the G1 phase (Figure S6D, Supporting Information). Additionally, transwell assays revealed that EHF effectively impeded RCC cell metastatic ability (Figure S6E,F, Supporting Information). These findings underscore the inhibitory impact of EHF on RCC progression.

**Figure 6:**
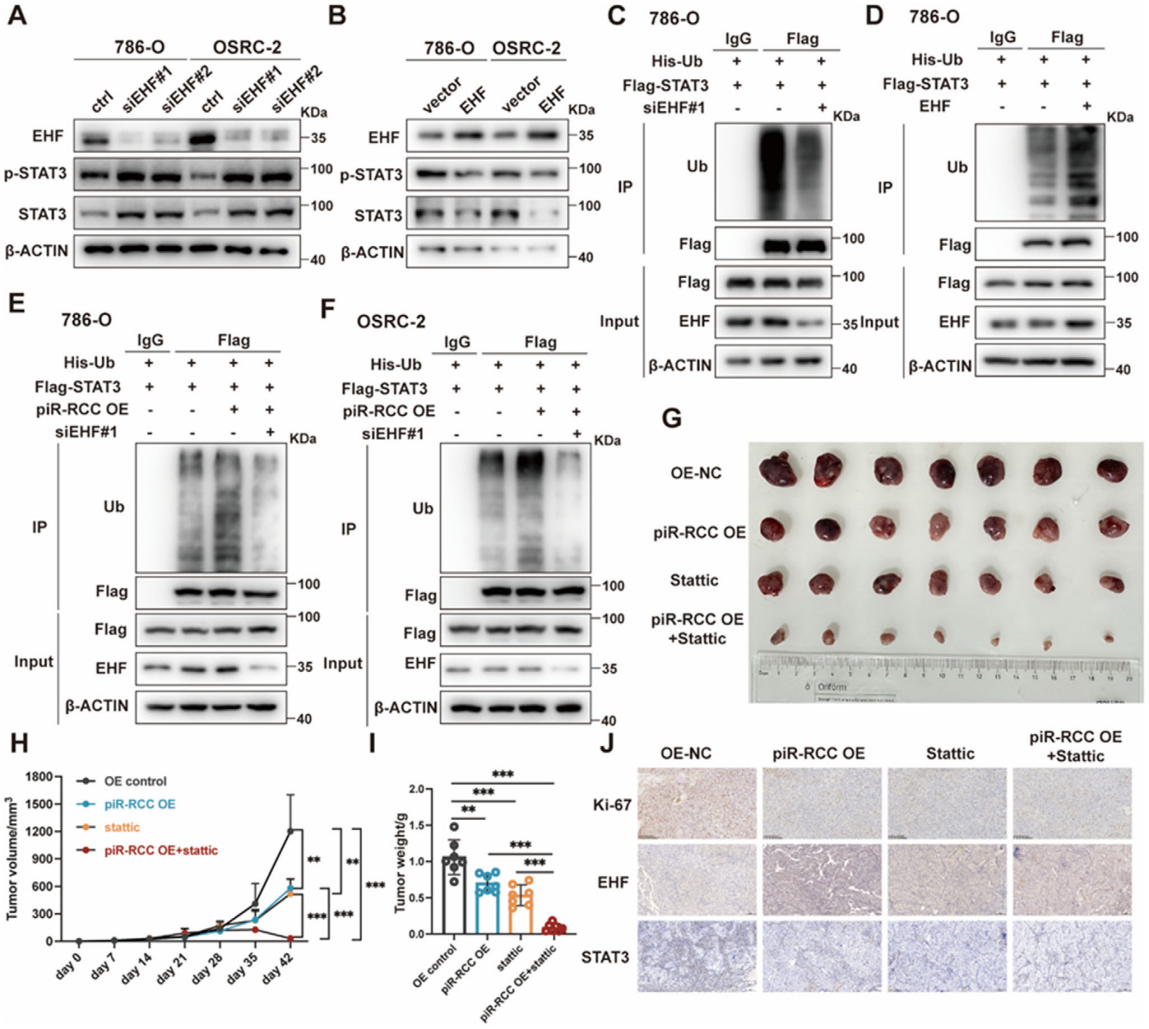
piR‐RCC restrains RCC progression via regulating EHF/STAT3 axis. A,B) STAT3 and p‐STAT3 protein expression levels in EHF knockdown or overexpression RCC cells. C,D) Western blotting assay was performed to detect the ubiquitination levels of STAT3 protein after EHF overexpression or knockdown in 786‐O cells. Ub, ubiquitin. E,F) Knockdown of EHF can abolish the effect of piR‐RCC overexpression on STAT3 ubiquitination levels. G–I) Images (G), volumes (H), and weights (I) of cell‐derived tumors from control or piR‐RCC overexpression OSRC‐2 cells treated with DMSO or static (10mg kg^−1^; every other day; intraperitoneal injection). J) Representative IHC staining images for Ki‐67, EHF, and STAT3 of OSRC‐2 cell–derived xenograft tumors. Scale bar, 200µm. Data are presented as mean ± SD; one‐way ANOVA was used for (H, I). ^*^ *p* < 0.05, ^**^ *p* < 0.01, ^***^ *p* < 0.001; ns, not significant.

Previous studies have shown that EHF can inhibit STAT3 signaling via promoting the ubiquitination of STAT3 proteins.^[^ ^41^ ^]^ Consequently, we assessed the levels of STAT3 and p‐STAT3 in RCC cells following EHF knockdown or overexpression. The results demonstrated that EHF knockdown elevated the STAT3 and p‐STAT3 levels, whereas EHF overexpression reduced their expression (**Figure** **6**A,B). piR‐RCC exhibited a similar trend in regulating STAT3 and p‐STAT3 expression levels (Figure 5D). Subsequent protein ubiquitination assays further indicated that silencing of EHF reduced STAT3 protein ubiquitination and degradation, while EHF overexpression promoted STAT3 protein ubiquitination and degradation (Figure 6C,D; Figure S6G,H, Supporting Information), aligning with previous research.

Subsequently, we delved into the involvement of EHF in the inhibitory effect of piR‐RCC on RCC progression. CCK‐8, colony formation, and transwell assays revealed that EHF knockdown could attenuate the inhibitory effect of piR‐RCC overexpression on RCC proliferation and metastasis (Figure S7A–E, Supporting Information). Meanwhile, western blot assays indicated that piR‐RCC overexpression reduced the expression of STAT3 and p‐STAT3, while EHF knockdown could reverse this effect (Figure S7F, Supporting Information). Protein ubiquitination assays also indicated that the increased ubiquitination level of STAT3 induced by piR‐RCC overexpression could be counteracted by EHF knockdown (Figure 6E,F). Totally, these data show that piR‐RCC inhibits RCC progression by regulating the EHF/STAT3 pathway.

### 2.7 Combination of piR‐RCC Overexpression and Stattic Exerts a Stronger Tumor Suppressor Effect

STAT3 is an important regulator in cancer; substantial evidence suggests that STAT3 is constitutively activated in many cancers, significantly contributing to tumor development and metastasis, and is associated with cancer severity and unfavorable prognosis.^[^ ^42^ ^]^ Given that piR‐RCC inhibits RCC progression by down‐regulating STAT3 expression, we investigated whether the combination of piR‐RCC overexpression and stattic (a STAT3 inhibitor) would synergistically inhibit RCC progression. Both CCK‐8 and xenograft animal models showed that either piR‐RCC overexpression or stattic treatment alone could hinder RCC proliferation. However, the combination of piR‐RCC overexpression and stattic exhibited an additive role in restraining RCC proliferation (Figure S7G, Supporting Information; Figure 6G–I). Moreover, we observed reduced expression of Ki‐67 (a tumor proliferation marker) and STAT3 in piR‐RCC overexpression or stattic treatment groups alone compared to the control group, with even lower expression in piR‐RCC overexpression and stattic combination treatment group (Figure 6J). Collectively, the combination of piR‐RCC overexpression and stattic exerts a synergistic effect in restraining RCC proliferation.

### 2.8 Efficient Delivery of piR‐RCC by Cancer Cell Membrane‐Coated Nanoparticles for Renal Cancer Therapy

**Figure 7:**
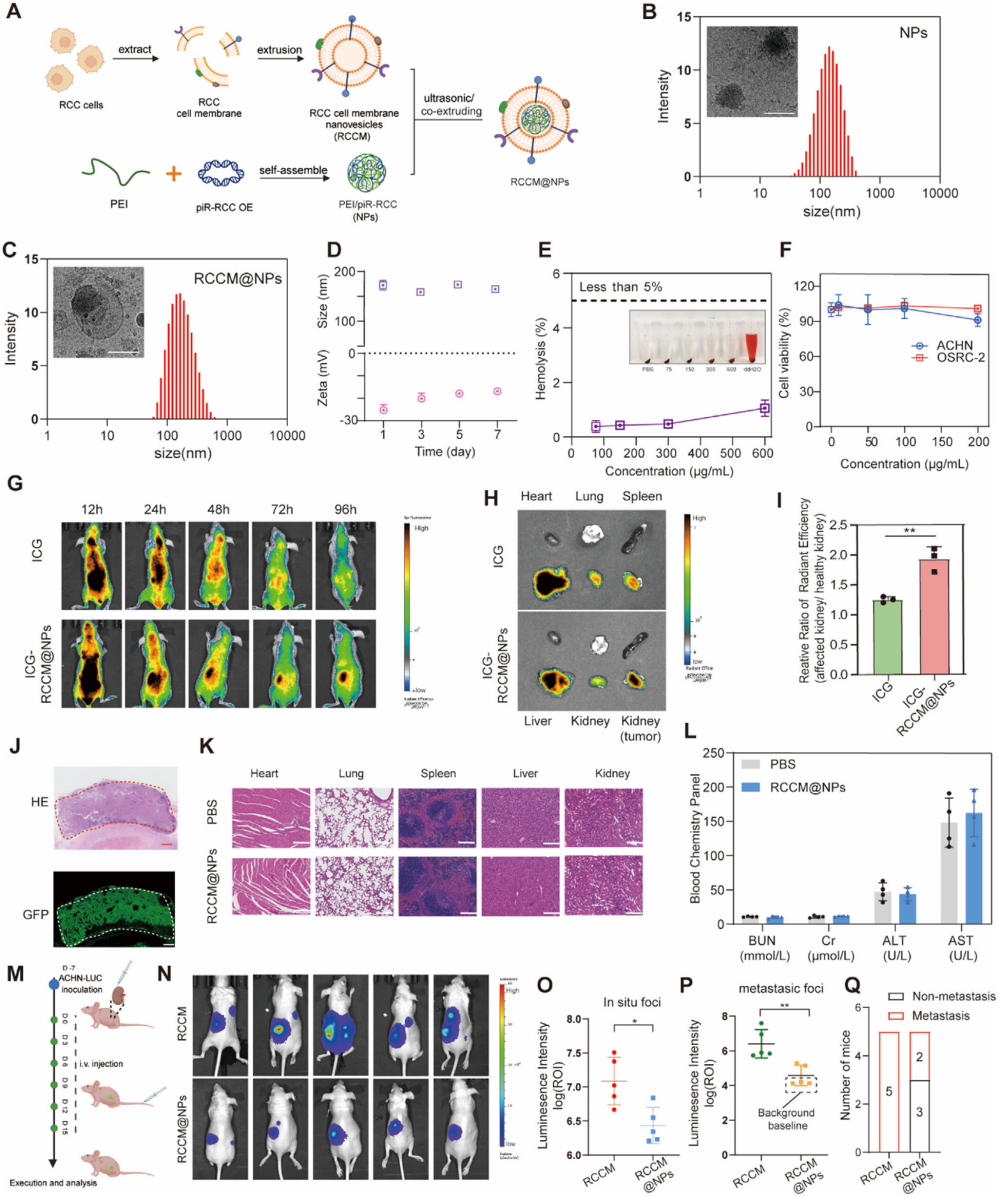
Characterization, targeting evaluation, therapeutic efficacy of RCCM@NPs. A) Schematic diagram of the preparation process of RCCM@NPs. B) Size and cryo–electron microscopy micrograph (inserted) of NPs. Scale bar = 100 nm. C) Size and cryo–electron microscopy micrograph (inserted) of RCCM@NPs. Scale bar = 100 nm. D) Stability of RCCM@NPs in 1×PBS, which includes the average particle size and zeta potential. E) Hemolysis analysis of RBC incubated with RCCM@NPs of various concentrations. Water and PBS were utilized as positive and negative controls, respectively. F) Cytotoxicity evaluation of different concentrations of RCCM@PEI on ACHN and OSRC‐2. G) Representative in vivo images of mice bearing orthotopic ACHN xenografts at different time intervals after intravenous injection of ICG or ICG‐RCCM@NPs. H) Representative ex vivo fluorescence images of ICG fluorescent dye accumulation in different organs 96 h after intravenous injection. I) Relative fluorescence intensity of compared to the non‐tumor‐bearing kidney in ICG and ICG‐RCCM@NPs groups, respectively (fluorescence intensity in tumor‐bearing kidney/non‐tumor‐bearing kidney) (mean ± SD, n = 3). J) H&E and immunofluorescence staining of kidney tissues in tumor‐bearing mice of RCCM@NPs group, the dashed box demarcates the tumor region. Scale bar, 500µm. K) H&E staining of tissue sections of major organs 21 days after injection (PBS or RCCM@NPs, every three days, intravenously). Scale bar = 200µm. L) Biochemical markers relevant to hepatic and kidney function (mean ± SD, n = 4). M) Illustrative depiction of the therapeutic experimental design and methodology. ACHN‐LUC cells were inoculated into the left kidney of BALB/c nude mice to establish an in situ tumor model. It also injected free RCCM and RCCM@NPs intravenously, respectively. N) Bioluminescent images showing primary foci and metastasis in nude mice (n = 5). O,P) Bioluminescent signal intensities (photons/s/cm2/sr) of primary foci (O) and metastasis foci (P) were quantified (for mice without tumor metastasis, the background baseline bioluminescence of their organs was used to substitute for metastatic foci intensity in statistical analyses). Q) Quantification of number of metastasis mice described in (N). Data are presented as mean ± SD, two‐tailed unpaired *t*‐test was used for (I, O, P). ^*^ *p* < 0.05, ^**^ *p* < 0.01.A and M created with BioRender.com, with permission.

In recent years, cell membrane cloaking technology, a top‐down biomimetic approach employing natural cell membranes as nanocarriers to facilitate the delivery of core materials, has shown tremendous potential in targeted drug delivery.^[^ ^43^, ^44^, ^45^, ^46^, ^47^ ^]^ On the other hand, gene therapy is an effective method for cancer treatment. Among these, polyethyleneimine (PEI) is one of the widely used gene carriers, condensing DNA into nanocomplexes to facilitate endocytosis.^[^ ^48^, ^49^ ^]^ Based on this, we designed a PEI/DNA delivery system coated with RCC cell membranes for efficient delivery of piR‐RCC plasmids, aiming at targeted therapy for RCC (**Figure** **7**A).

The biomimetic drug delivery system consists of two parts: internal cationic polymers PEI (gene‐carrying core) and a RCC cell membrane coating (functional layer for targeting renal carcinoma). Here, PEI self‐assembles with negative‐charged piR‐RCC overexpression plasmids via electrostatic interactions to form complexes constituting PEI/piR‐RCC core (NPs). Simultaneously, RCC cell membrane vesicle (RCCM) was obtained through ultrasonic or extrusion. Subsequently, RCCM was encapsulated on the surface of the PEI/piR‐RCC plasmids through ultrasound/extrusion, to acquire the final biomimetic nanomedicine RCCM@NPs.

To successfully prepare the safe and efficient gene vector, we investigated the DNA condensation capability of PEI at different nitrogen/phosphate molar (N/P) ratios by the gel retardation assay; the result indicated that PEI could fully condense plasmid DNA (pDNA) in terms of N/P ratio higher than or equal to 8 (Figure S8A, Supporting Information). Based on this, we selected the N/P ratio of 8 for subsequent experiments. The resulting RCCM@NPs exhibited an average diameter of ≈160.7±11.9 nm, and its zeta potential (−16.6±2.4 mV) was close to that of RCCM vesicles (Figure 7B,C; Figure S8B, Supporting Information), and the results of cryo‐electron microscopy indicated successful surface coating with cell membrane (Figure 7C). Coomassie brilliant blue staining demonstrated a similar total protein profile between RCCM@NPs and cancer cell membrane, confirming the effective retention of membrane proteins from source cell membranes on RCCM@NPs after coating (Figure S8C, Supporting Information).

The hydrodynamic diameter of RCCM@NPs stored in PBS was monitored for 5 days, indicating its long‐term stability (Figure 7D). Good biocompatibility is an important prerequisite for its translation into clinical applications. Hence, the toxicity and biosafety of nanoparticles were comprehensively evaluated in vitro. RCCM@NPs exhibited good blood compatibility, with a hemolysis rate still below 5% even at concentrations as high as 600 µg/mL (Figure 7E). The cytotoxicity of nanoparticles was detected using the CCK8 assay. RCCM@NPs had almost no cytotoxicity, with a cell survival rate of ≈90% even at a concentration of 100 µg/mL (Figure 7F; Figure S8D, Supporting Information).

To assess the biodistribution and targeting ability of nanoparticles in mice, we established a renal orthotopic tumor model by orthotopically injecting ACHN cells into the left kidney of nude mice, and then intravenously injected Indocyanine Green (ICG)‐labeled RCCM@NPs via tail vein into the nude mice. Then, fluorescence imaging was conducted to monitor the real‐time distribution of RCCM@NPs and their accumulation in tumors. At 24 h post‐injection, significant enrichment was observed at the tumor site. 72 h post‐injection, the RCCM@NPs group still retained a strong signal at the left kidney (Figure 7G). Additionally, in the RCCM@NPs‐treated group, the left kidney displayed significantly higher fluorescence signals compared to the non‐tumor‐bearing right kidney, which was also confirmed in *ex vivo* imaging (Figure 7H,I; Figure S8E, Supporting Information). This indicates that our nanomedicine accumulates in the tumor site rather than in normal kidney tissue. Additionally, piR‐RCC overexpression plasmid expressed GFP fluorescence, and immunofluorescence and H&E staining of tissue sections from the left kidney of the RCCM@NPs‐treated group indicated the predominant accumulation of the nanomedicine in the tumor tissue (Figure 7J). These findings might be attributed to the prominent long‐circulation capability and targeting specificity of RCCM@NPs. Fluorescence signals of ICG were also detected in other major organs, primarily in the liver, possibly due to the direct injection of nanomedicine into the bloodstream and subsequent reticuloendothelial system clearance in the liver (Figure S8F, Supporting Information). Overall, the experimental data demonstrate that RCCM@NPs exhibits higher accumulation at the tumor site, displaying excellent tumor‐targeting capabilities.

The biocompatibility of RCCM@NPs nanoparticles in vivo was studied using blood biochemistry. These parameters showed no significant differences compared to the PBS control group, indicating negligible hematological toxicity of RCCM@NPs in vivo. Furthermore, histological images of major organ samples did not reveal significant signs of tissue damage or inflammation, suggesting that the administration of nanoparticles did not induce systemic toxicity (Figure 7K,L).

Subsequently, to evaluate the in vivo therapeutic efficacy of RCCM@NPs, we orthotopically implanted luciferase‐labeled ACHN cells under the left kidney capsule of nude mice. One week after inoculation, the nude mice were randomly divided into two groups (n = 5), and were intravenously administered with RCCM and RCCM@NPs via tail vein, with treatments repeated every three days for a total of six doses (Figure 7M). Bioluminescence imaging revealed that at the end of the experiment, the nanoparticle group, as compared to free RCCM, inhibited tumor progression at the orthotopic lesion site and reduced metastatic occurrences (Figure 7N‐Q). Taken together, RCCM@NPs exhibits good tumor targeting ability, therapeutic efficacy, and biocompatibility.

**Figure 8:**
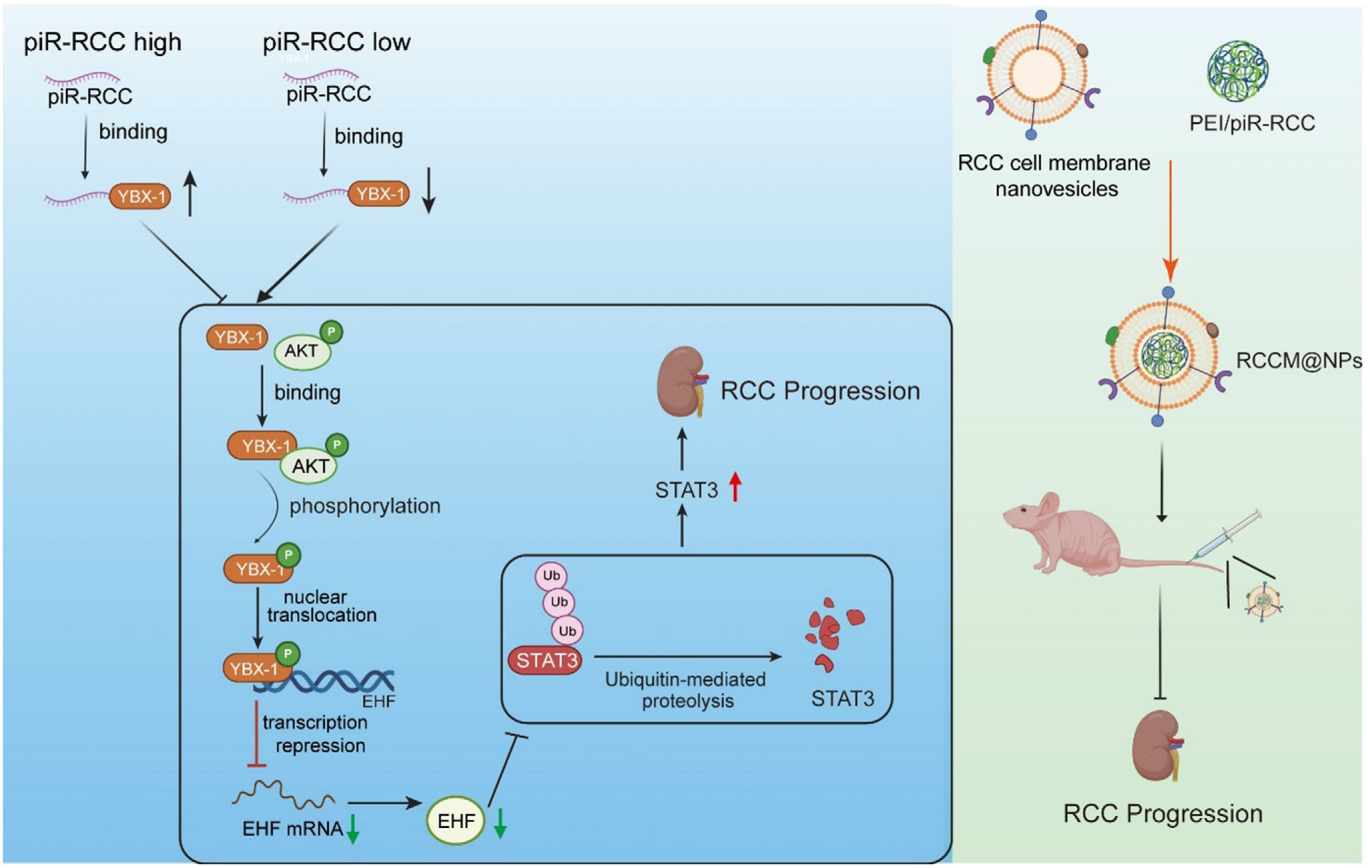
Schematic of the proposed molecular mechanisms for piR‐RCC involvement in RCC progression. Image created with BioRender.com, with permission.

## 3 Discussion

As only 2–3% of the human genome is transcribed into proteins, non‐coding RNAs (ncRNAs) have gained attention for their pivotal roles in cellular function and disease mechanisms.^[^ ^50^ ^]^ Among these, piRNAs, a burgeoning family of short ncRNAs, have surpassed 6.9 × 10^6^ identified members in the human genome according to current database annotations, indicating their potential regulatory roles.^[^ ^51^ ^]^

piRNAs, initially discovered in animal germ cells, play a pivotal role in germ cell development. Their classical function involves silencing transposable elements at both transcriptional and post‐transcriptional levels, thereby maintaining genomic integrity.^[^ ^5^ ^]^ Beyond germline cells, piRNA pathways have been observed in non‐germline cell contexts. Dysregulation of piRNAs has been implicated in various cancers, including breast, lung, and liver cancers, indicating a broader impact beyond their original germ cell role.^[^ ^50^, ^52^, ^53^, ^54^, ^55^ ^]^ As for piRNAs in renal cancer, piRNAs can act as tumor suppressors or oncogenes in RCC. PiR‐31115 may activate epithelial‐mesenchymal transition (EMT) via the PI3K/AKT signaling pathway, and then promote mobilityand invasiveness in RCC.^[^ ^56^ ^]^ Besides, Zhang et al. demonstrated that piR‐1742 promoted renal cell carcinoma malignancy by regulating USP8 stability.^[^ ^57^ ^]^ On the other hand, piRNAs have the potential to serve as prognostic biomarker in RCC.^[^ ^58^, ^59^ ^]^

The function of piRNA in cancers remains ambiguous. Prior research indicates that piRNAs can influence post‐transcriptional gene regulation by directly binding to target mRNA, resulting in mRNA degradation, or associating with PIWI or other proteins, modifying the expression or activity of the target protein to produce various biological activities. Recently, Wang et al. demonstrated that piRNA PROPER interacts with YTHDF2 and YBX3 to constitute the piRNA‐induced silencing complex (pi‐RISC), therefore suppressing translation and facilitating prostate cancer progression.^[^ ^60^ ^]^ YBX‐1 and YBX‐3 both belong to the YBX family. Similar to the aforementioned studies, in our research, piRNA directly binds to YBX‐1, disrupting its phosphorylation and affecting downstream functions. In addition, other studies have reported that piRNA targets YBX‐1 mRNA, thereby inhibiting triple‐negative breast cancer.^[^ ^61^ ^]^ Based on the above evidence, the interaction between piRNAs and the YBX protein family may exert diverse effects in different types of tumors.

Aberrant phase separation is now considered a potential mechanism in various biological processes, with liquid‐liquid phase separation (LLPS) being a physical‐chemical process. Our work demonstrates that piR‐RCC inhibits the binding of YBX‐1 and p‐AKT by interacting with YBX‐1, consequently disrupting the phosphorylation level of YBX‐1. Previous research indicates that, apart from mature rRNA, most RNAs influence phase separation to some degree, and YBX‐1 demonstrates phase separation properties.^[^ ^12^, ^62^, ^63^, ^64^ ^]^ Lu et al. and Liu et al. have respectively proposed that lncRNA and circRNA can drive the phase separation of YBX‐1.^[^ ^65^, ^66^ ^]^ Hence, at times, we speculate that in a more detailed mechanistic context, piR‐RCC might also potentially influence downstream pathways by inducing YBX‐1 phase separation. However, this is purely speculative and requires further experimental exploration.

Moreover, piRNAs can easily exit the cell through extracellular vesicles (EVs), where they are less prone to degradation.^[^ ^6^, ^67^, ^68^, ^69^, ^70^ ^]^ Consistent with previous studies, we found that piR‐RCC is relatively stable in exosomes (unpublished). Given the stability of piRNAs and their relative abundance in exosomes, exploring the circulating piRNAs profile in cancer will be particularly interesting. Their levels in body fluids could potentially provide diagnostic potential as non‐invasive biomarkers.

Finally, we employed a biomimetic nanoparticle system to deliver piR‐RCC. Nanoparticle delivery systems are primarily categorized into conventional nanoparticle delivery systems and biomimetic nanoparticle delivery systems.^[^ ^28^, ^29^, ^71^, ^72^, ^73^ ^]^ The latter harnesses biological membranes (such as red blood cells, tumor cells, exosomes, and liposomes) to encapsulate nanoparticles, aiming to impart the inherent properties of cell membranes to the nanoparticles while retaining their intrinsic physicochemical characteristics. This approach results in lower immunogenicity, excellent biocompatibility, prolonged in vivo circulation time, and outstanding target specificity.^[^ ^28^, ^29^, ^72^, ^73^ ^]^ Here, we utilized renal cancer cell membrane‐coated nanoparticles to target the delivery of piR‐RCC in orthotopic kidney tumor model, offering a translational avenue for piRNA‐based gene therapy (**Figure** 8.). Previous studies suggested that adhesion molecules or receptors play crucial roles in mediating the homing of tumor cells.^[^ ^29^, ^71^ ^]^ Therefore, identifying more specific adhesion molecules or receptors targeting renal tumors may enable more controlled, precise, and streamlined targeting.

## 4 Conclusion

In conclusion, our study identifies piR‐RCC as a significant regulator that suppresses progression of renal cell cancer. Mechanistically, piR‐RCC directly binds to YBX‐1, thereby interfering with the interaction between YBX‐1 and p‐AKT, which affects the phosphorylation level of YBX‐1, leading to the inhibition of YBX‐1 nuclear translocation. As YBX‐1 acts as a transcriptional repressor on EHF, piR‐RCC promotes the transcription of EHF regulated by YBX‐1, affecting its downstream pathways and mediating the inhibition of RCC progression. Finally, we construct a biomimetic nanoparticle system to achieve the renal cancer‐specific targeted delivery of piR‐RCC, and offer a promising therapeutic avenue for RCC.

## 5 Experimental Section

### Clinical Specimens

RCC specimens in the cohort were obtained from Sir Run Run Shaw Hospital, School of Medicine, Zhejiang University, and approved by the ethics committee of Sir Run Run Shaw Hospital, School of Medicine, Zhejiang University (20210714‐59). Informed consent was assigned from the patients. The detailed clinical characteristics of RCC patients are shown in Table S1 (Supporting Information).

### Cell Lines and Cell Culture

The human RCC cell lines (786‐O and OSRC‐2) were purchased from Cell Bank of Shanghai Institute of Biochemistry and Cell Biology (SIBCB). RCC cell lines were cultured in RPMI‐1640 medium (Cienry, China) supplemented with 10% FBS (Cellmax, China). All cells were maintained at 37 °C with 5% CO2.

### piRNA Sequencing

Three paired ccRCC and adjacent normal tissue samples were acquired from surgical specimens at the Department of Urology, Sir Run Run Shaw Hospital. Total RNA from each sample was quantified using a NanoDrop ND‐1000 instrument and was used to prepare the sequencing library in the following steps: 1) 3′‐adapter ligation; 2) 5′‐adapter ligation; 3) cDNA synthesis; 4) PCR amplification; 5) size selection of 142–153bp PCR amplified fragments. The libraries were denatured into single‐strand DNA molecules, amplified in situ, and subsequently sequenced on the Illumina NextSeq500 according to the manufacturer's guidelines.

### RNA Sequencing and Screening Workflow

Total RNA was extracted with TRIzol reagent (CWBio) following the manufacturer's instructions. Following the extraction of total RNA, mRNA was isolated from total RNA utilizing Dynabeads Oligo (dT) (Thermo Fisher, CA, USA). Following purification, the mRNA was fragmented into short fragments. Then, the RNA was used for sequencing library preparation (NEB, USA). Sequencing data was collected using the Illumina Novaseq 6000 (LC‐Bio Technology CO., Ltd., Hangzhou, China) following the vendor's recommended protocol.

The screening criteria for differentially expressed genes were: absolute log2(fold change) ≥ 1 and adjusted p‐value < 0.05. Further screening workflow for the integrated analysis of piR‐RCC RNA‐seq and YBX‐1 RNA‐seq data: 1) showing differential expression in the TCGA‐KIRC dataset between tumor with normal tissue; 2) having read counts greater than 10 in both piR‐RCC and YBX‐1 RNA‐sequencing data.

### RNA Extraction and Reverse Transcription‐Quantitative Real‐Time PCR (RT‒qPCR) Assay

Total RNA from RCC tissues and cells was extracted using TRIzol reagent (CWbio, China) according to the manufacturer's instructions. The miRNA 1st strand cDNA synthesis kit (MR101, Vazyme) was used to amplify the specific piRNA with piRNA stem‐loop primers. Reverse transcription was carried out using the HiFiScript cDNA Synthesis Kit (CWbio) according to the manufacturer's instructions. qRT‐PCR analysis was performed using the SYBR Green (CWbio) with GAPDH as the internal control on LightCycler 480 System (Roche). All primers were synthesized by Tsingke Biotech (China). The relative expression of piRNA or mRNA was determined using 2^–ΔΔCt^ method. The detailed primer information is shown in Table S2 (Supporting Information).

### Isolation of Cytoplasmic and Nuclear Fractions

Cytoplasmic and nuclear RNA isolation was performed using the Cytoplasmic & Nuclear RNA Purification Kit (Norgen Biotek Corp, Canada) in accordance with the manufacturer's guidelines. GAPDH and U6 were selected as reference for the cytoplasm and nucleus, respectively. The primer pairs employed in this study are detailed in Table S2 (Supporting Information).

### Cell Transfection

The piR‐RCC inhibitors, piR‐RCC mimics, YBX‐1 siRNAs and EHF siRNAs were purchased from GenePharma (China). The plasmid of GFP‐piR‐RCC (only used in the RCCM@NPs) and YBX‐1(S102A) was purchased from Loche Biomedical Technology (China), and other plasmids and lentivirus in this study were obtained from Geenchem (China). RNAimax (Invitrogen) was utilized for siRNAs, piR‐RCC inhibitors, and piR‐RCC‐mimics transfection. And Lipofectamine 3000 (Invitrogen) were employed for plasmids transfection. Puromycin (1 µg mL^−1^) was used to select RCC cells which were transfected with piR‐RCC knockdown or overexpression lentivirus. The siRNAs sequences are shown in Table S3 (Supporting Information).

### Cell Proliferation Assay

For cell counting kit‐8 (CCK‐8) assay, the transfected RCC cells were seeded into the 96‐well plates at a density of 1000 cells per well. After 1, 2, 3, 4, 5 days, the CCK‐8 reagent (Dojindo Laboratories) was added to each well. After a 2‐h incubation, the absorbance at 450 nm was measured by Multiskan FC Microplate Photometer (Thermo Scientific).

For colony formation assay, the transfected RCC cells were seeded into the 6‐well plates at a density of 1000 cells per well. After 10–14 days, the cells were fixed by 4% paraformaldehyde, stained with 0.3% crystal violet, photographed using SYSTEM GelDoc XR (Bio‐rad).

For EdU assay, the transfected RCC cells were seeded into the 96‐well plates at a density of 5000 cells per well. Then, the RCC cells were incubated with 20 µM EdU (Meilunbio, China) overnight. The proliferation ability of RCC cells was then detected using EdU Cell Proliferation Kit (Meilunbio) according to the manuscript instructions. The images were captured by fluorescence microscope (Olympus).

### Wound Healing Assay

The transfected RCC cells were seeded into the 6‐well plates. Once the cell confluence reached 90%, a 200 µL pipette was used to scratch the cells to create a straight wound. Then, the wound was photographed at 0 and 24h using microscope.

### Transwell Migration and Invasion Assay

For transwell migration assay, a total of 1 × 10^4^ transfected RCC cells were suspended in 200 µL FBS free medium and seeded into the upper chamber of 8‐µm pore inserts (Corning). Then, 200 µL medium containing 10% FBS was added into the lower chamber. After 24 h, the migrated cells were fixed and stained with 0.3% crystal violet.

For transwell invasion assay, a total of 3×10^4^ transfected RCC cells were added into the upper chamber, which was coated with Matrigel (Corning). And subsequent procedures were identical to those of the transwell migration assay.

### Flow Cytometry Assay

For cell cycle assay, the RCC cells (transfected with piR‐RCC inhibitors or mimics) were collected and washed twice. Then, the cells were stained with PI staining solution (Multi Science). After incubation for 20 min, the cell cycle of RCC cells was detected using flow cytometry.

For apoptosis assay, the transfected RCC cells were collected and washed for twice. Then, the cells were stained with annexin V‐FITC and PI staining solution (Multi Science). After incubation for 5 min, the apoptosis rate of RCC cells were determined using flow cytometry.

### Western Blotting

Total proteins from tissues and cells were extracted using RIPA lysis buffer (FDbio). The proteins were separated by SDS‐PAGE and then transferred to the 0.2 µm PVDF membrane (Bio‐Rad). The membrane was blocked by 5% nonfat milk and subsequently incubated with the primary antibodies at 4 °C overnight. The next day, the membranes were washed for three times and incubated with the secondary antibodies (Jackson ImmunoResearch). Finally, the membranes were visualized by ECL kit (FDbio). The antibodies information is shown in Table S4 (Supporting Information).

### Fluorescence In Situ Hybridization (FISH) Assay

For the FISH assay, the piR‐RCC‐specific probe was designed and produced by GenePharma. About 2×10^5^ RCC cells were seeded into 24‐well plates. Then, the FISH assay was performed using FISH kit (GenePharma) according to the manufacturer's instructions. Finally, the stained cells were photographed using confocal microscopy Olympus fv3000 (Olympus). The piR‐RCC FISH probe information is shown in Table S5 (Supporting Information).

### Chromatin Immunoprecipitation (ChIP) Assay

A total of 2×10^7^ RCC cells were used for ChIP assay. ChIP assay was performed using the SimpleChIP Enzymatic Chromatin IP Kit (CST, USA) according to the manufacturers’ instructions. Briefly, cells were cross‐linked with 1% formaldehyde and lysed using ChIP lysis buffer. The lysate was sonicated to shear DNA. YBX‐1 antibodies and IgG were employed for exploration. The precipitated DNA was detected by qPCR. The specific ChIP primers information is shown in the Table S2 (Supporting Information).

### Immunofluorescence (IF) Assay

About 2 × 10^5^ RCC cells were seeded into 24‐well plates. After adherence, the RCC cells were washed and fixed for 15 min. After washed for 3 times, the RCC cells were treated with 0.2% Triton X‐100 for 10 min. Then, the cells were washed with PBS and blocked using 5% BSA for an hour. Then, the cells were incubated with primary antibodies YBX‐1 (1:200) at 4 °C overnight. The next day, the RCC cells were washed for 3 times and incubated with the fluorescent secondary antibodies (Invitrogen) for an hour at room temperature. Finally, the RCC cells were stained with DAPI for 10 min. The stained cells were photographed using confocal microscopy (Olympus fv3000) or a fluorescence microscope (Olympus).

### RNA Pulldown Assay

The RNA pulldown assay was performed based on the previous publication.^[^ ^74^ ^]^ Briefly, the biotin‐labeled piR‐RCC probe was synthesized by Tsingke (China). A total of 2 × 10^6^ RCC cells were lysed in lysis buffer (50 mM Tris‐HCl PH = 7.4, 150 mM NaCl, 2mM MgCl_2_, 1% NP40, protease inhibitors and RNase inhibitors) at 4 °C for 30 min. Then, the cell lysate was incubated with the piR‐RCC probe (Table S6, Supporting Information) at 4 °C for 30 min, followed by combining with streptavidin agarose beads (Invitrogen) for 2 h. After being washed 10 times, the RNA‐protein complex was collected and detected by western blot and mass spectrometry.

### RNA Immunoprecipitation (RIP) Assay

The RIP assay was performed using Magna RIP Kit (Millipore, USA) according to the manufacturers’ instructions. Briefly, a total of 2 × 10^7^ RCC cells were lysed in RIP lysis buffer. Then, the supernatant was incubated with primary antibodies or IgG and magnetic protein A/G beads at 4 °C overnight. The following day, the antibodies‐RNA complex was washed for 5 times and digested by protease K. Finally, the immunoprecipitated RNA was purified and detected by RT‐qPCR.

### Co‐Immunoprecipitation (Co‐IP) Assay

A total 1 × 10^6^ RCC cells were lysed in IP lysis buffer (FDbio) at 4 °C for 30 min. Then, the cell lysate was incubated with primary antibodies at 4 °C overnight. The following day, Protein G Sepharose 4 Fast Flow resins (cytiva) were added into the mixture for 3 h. After being washed 5 times, the immunoprecipitated protein was detected by western blot.

### Molecular Docking

The protein‐resolved structures of YBX‐1 (PDB ID: 6KTC) were obtained from the PDB database (http://www.rcsb.org/), and the m5C RNA as well as small‐molecule ligands of the receptor were deleted and pre‐processed for hydrogenation, etc., in PyMOL 2.4 software. The molecular structure of piR‐RCC was predicted by the 3dRNA database (http://biophy.hust.edu.cn/new/).^[^ ^75^ ^]^ Molecular docking was performed using HDOCK SERVER (version 2023‐09‐09).^[^ ^76^ ^]^

### Animal Assays

The animal experimental protocols were approved by the ethics committee of Sir Run Run Shaw Hospital, School of Medicine, Zhejiang University (SRRSH202107081).

For xenograft animal model, a total of 2 × 10^6^ stably transfected OSRC‐2 cells were inoculated subcutaneously into the right flank of each 4‐week‐old female BALB/c nude mice. Tumor size was measured every 7 days, and the tumor volume was calculated by the equation:

(1) $$\mathrm{Volume}=\mathrm{length}\;\times \;\mathrm{widt}h^{2}/2$$

After 6 weeks, the mice were euthanized, and the xenograft tumors were excised and weighed.

For renal orthotopic implantation model, 2 × 10^6^ ACHN cells were suspended in a 50 µL mixture of PBS and Matrigel (1:1 ratio) and then injected under the renal capsule of 4‐week‐old BALB/C Nude mice. After an appropriate time period (5 weeks for the effect of piR‐RCC on tumor metastasis in vivo; 8 weeks for therapeutic efficacy of RCCM@NPs), mice were anesthetized, and tumor growth and metastasis were detected using an in vivo imaging system (IVIS).

### Preparation of PEI/pDNA Polyplex

Polyethylenimine (PEI, Mw = 40K) was purchased from FuShenBio Co., Ltd. (Shanghai, China). PEI was dissolved in HEPES buffer (pH7.4, 10mM) at 1mg mL^−1^. The needed concentrations of PEI solution according to the designated N/P ratios, defined as the molar ratio of nitrogen atoms in the polymer to phosphate atoms in the plasmid DNA (pDNA). Then DNA plasmid (GFP‐labeled) solution and the PEI solution are mixed, vortexed for 10 s, and then incubated at room temperature for 20 min.

### Preparation of RCCM@NPs

The ACHN cells was isolated using a cell scraper. Subsequently, the collected cells were washed with chilled PBS to remove residual culture media. Afterward, the obtained cell pellet will be resuspended in Chemical A from the Membrane and Cytoplasmic Protein Extraction Kit (Beyotime Biotechnology) containing PSMF (Beyotime Biotechnology), and incubated on ice for 15 min. Following the manufacturer's instructions, cell disruption was achieved by subjecting the mixture to three cycles of freeze‐thaw. The resulting mixture was further centrifuged at 700 × g for 10 min at 4 °C to obtain the supernatant, followed by centrifugation at 14 000 × g for 20 min at 4 °C to collect the ruptured cell membrane precipitate. The cell membrane was then extruded through 400 and 200 nm polycarbonate porous membranes (Whatman) by an Avanti mini extruder (Avanti), and the resulting vesicles were coated onto NPs by sonication or co‐extruding vesicles and cores through a 200 nm polycarbonate membrane to yield RCCM@NPs.

### Characterization of RCCM@NPs

The diameter and zeta potential of nanoparticles were measured by Malvern Zetasizer (Malvern Instruments, UK). The morphology was observed via cryo‐electron microscopy (Cryo‐EM) (Thermo Scientific, Talos F200C 200‐kV).

### Hemolysis Assay

Blood was sampled from mice and collected into heparin sodium pre‐treated tubes, and red blood cells (RBCs) were separated from serum through centrifugation and washing. The RBCs were then incubated with RCCM@NPs in PBS at 37 °C for 2 h. After the incubation, the mixture was centrifuged (860 g, 5 min), and the absorbance of the supernatant was measured using a microplate spectrophotometer at a wavelength of 541 nm. Water and PBS were employed as the positive and negative controls, respectively.

(2) $$\begin{array}{ccc} \mathrm{Hemolysis}\;(\%) & = & (\mathrm{OD}\;\mathrm{sample}\;-\;\mathrm{OD}\;\mathrm{negative}\;\mathrm{control})/\\ & & (\mathrm{OD}\;\mathrm{positive}\;\mathrm{control}\;-\;\mathrm{OD}\;\mathrm{negative}\;\mathrm{control})\times 100 \end{array}$$

### In Vitro Cytotoxicity Assay

The ACHN or OSRC‐2 cells were seeded in the 96 well plates and treated with different formulations or different concentration, including PBS, RCCM, RCCM@NPs. After incubation of 24 h, the CCK‐8 was added to keep 2 h and measure the cell viability by CCK‐8 assay.

### In Vivo the Ability of Tumor Targeting

For renal orthotopic implantation model, 2 × 10^6^ ACHN cells were suspended in a 50 µL mixture of PBS and Matrigel (1:1 ratio) and then injected under the renal capsule of 4‐week‐old BALB/C Nude mice. Following a 3‐week period, mice were anesthetized, and tumor growth and metastasis were detected using an in vivo imaging system (IVIS). To evaluate the in vivo localization of the nanoparticles, renal orthotopic implantation mice were intravenously injected with ICG and ICG‐labeled RCCM@NPs solutions into the tail vein. Biodistribution analysis of ICG was conducted using a 710‐nm excitation wavelength and a 785‐nm filter at 12, 24, 48, 72, 96 h post‐injection. At the end point of the experimental design time, the heart, lung, spleen, liver and kidneys of mice were harvested for in vitro imaging photography to detect enrichment in different organs.

### Biocompatibility Evaluation

Healthy mice were administered intravenously with RCCM@NPs or PBS a total of six times over a period of 21 days. After 21 days, blood samples were collected serum biochemistry analysis, including alanine aminotransferase (ALT), aspartate transaminase (AST), blood urea nitrogen (BUN), and creatinine (CRE). Additionally, major organs (heart, liver, spleen, lung, and kidney) were harvested and fixed in 4% paraformaldehyde (PFA) for H&E staining.

### In Vivo Therapeutic Efficacy RCCM@NPs

ACHN‐LUC (≈2 × 10^6^ cells) were mixed with 50 µL PBS/Matrigel mixture at 1:1 (v/v) and injected under the renal capsule of 4‐week‐old BALB/C Nude mice. After 7 days, the mice were randomly divided into two groups and injected with RCCM and RCCM@NPs (piR‐RCC overexpression plasmid dose: 0.5 mg kg^−1^) every three days for 6 times via tail vein. At the end of experiment, all mice were sacrificed to obtain the tumors further analysis including H&E and immunofluorescent experiment.

### Public Data Processing

The GEPIA online database (http://gepia.cancer‐pku.cn/) was used to analyzed the expression of EHF in RCC tumors and normal tissues.^[^ ^77^ ^]^ Datasets GSE183496 was obtained from GEO portal (https://www.ncbi.nlm.nih.gov/gds/).

### Statistical Analysis

Data were presented at mean ± standard deviation (SD). The Figures were plotted by GraphPad Prism 8 (GraphPad Software). The Wilcoxon matched‐pairs signed‐rank test was used to calculate the differential expression in clinical samples. The two‐tailed Student's t test was performed to evaluate the differences between two groups, and one‐way ANOVA was used for comparisons involving more than two groups. The p value < 0.05 was considered to be statistically significant.

### Ethical Statement

Ethics approval was obtained from Ethics Committee of SRRSH, and Informed consent was obtained from all patients involved in the study. All procedures in the in vivo experiment were conducted in accordance with institutional guidelines and approved by the Animal Research Ethics Committee of Zhejiang University.

## Conflict of Interest

The authors declare no conflict of interest.

## Author Contributions

R.W., F.L., and Y.L. contributed equally to this work. Conceptualization was carried out by G.H.L., L.F.D., and M.C.W.; Methodology by R.Y.W., F.L., and Y.D.L.; Investigation by R.Y.W., Y.D.L., Z.Y.L., W∖.Q.L., Z.H.X., Z.W∖.Z., Y.L., H.H.L., Y. Li, Z.N.S., and Y.N.C.; Formal Analysis by R.Y.W., X.D.M., and F.L.; Data Curation by R.Y.W. and L.F.D.; Visualization by R.Y.W., F.L., and Y.L.; Writing – Original Draft by R.Y.W., F.L., and Y.D.L.; Writing – Review and Editing by L.Q.X., W∖.Q.L., Z.Y.L., Y.N.C., and L.F.D.; and Supervision by G.H.L., L.F.D., and M.C.W.

## Supporting information



Supporting Information

## Data Availability

The piRNA sequencing of paired renal cell carcinoma tissues and adjacent normal tissues have been deposited in National Genomics Data Center, China National Center for Bioinformation / Beijing Institute of Genomics, Chinese Academy of Sciences (GSA‐Human: HRA009136) that are available upon reasonable request. The RNA‐seq data between piR‐RCC knockdown vs control, the RNA‐seq data between siYBX‐1 vs control in RCC cells have been deposited in the aforementioned database under the accession number: HRA010737 and HRA010731.
