# Supplementary material for: piR‐RCC Suppresses Renal Cell Carcinoma Progression by Facilitating YBX‐1 Cytoplasm Localization
Source: Adv Sci (Weinh). 2025 May 24;12(30):e14398. doi: 10.1002/advs.202414398 (PMC12376648; doi:10.1002/advs.202414398)

Supporting Information

**piR-RCC suppresses renal cell carcinoma progression by facilitating YBX-1 cytoplasm localization**

Ruyue Wang, Fan Li, Yudong Lin, Zeyi Lu, Wenqin Luo, Zhehao Xu, Ziwei Zhu, Yi Lu, Xudong Mao, Yang Li, Zhinian Shen, Haohua Lu, Yining Chen, Liqun Xia, Mingchao Wang*, Lifeng Ding*, Gonghui Li*


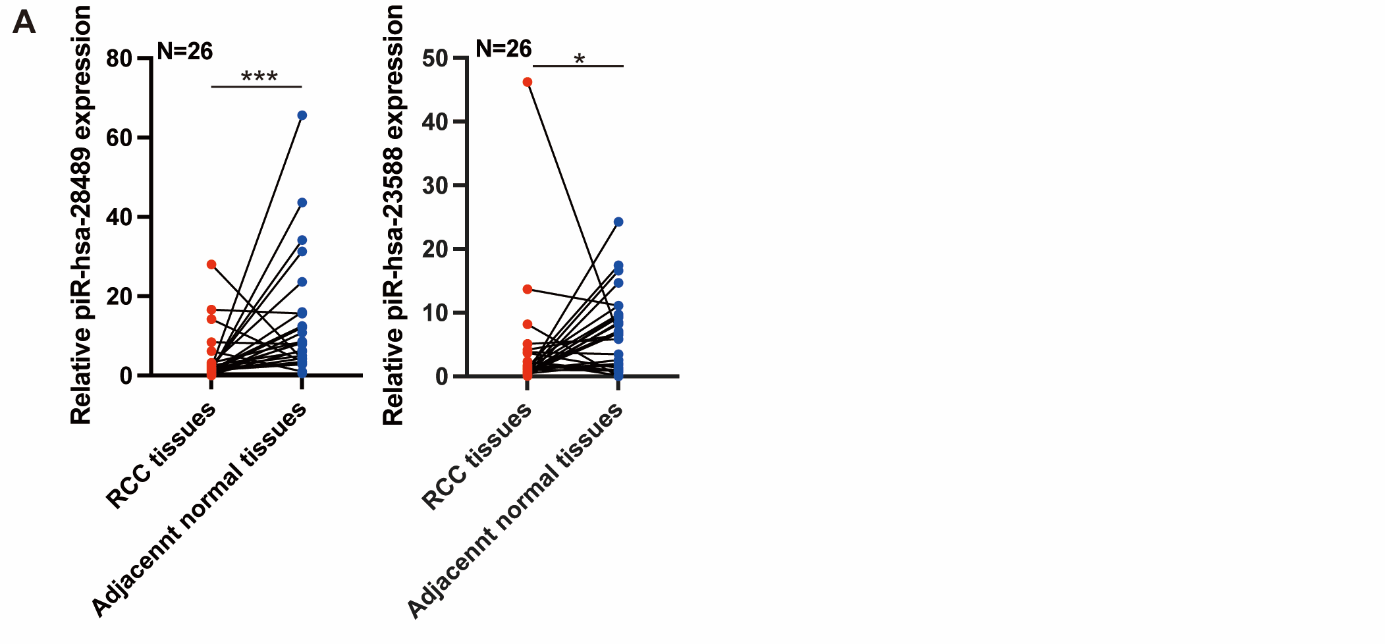
**Figure S1. The expression of piRNAs in RCC tissues.**

**(A)** The qRT-PCR assays results showing piR-hsa-28489 and piR-hsa-23588 level in RCC specimen and adjacent normal tissues from SRRSH cohort. Paired t test for (A).


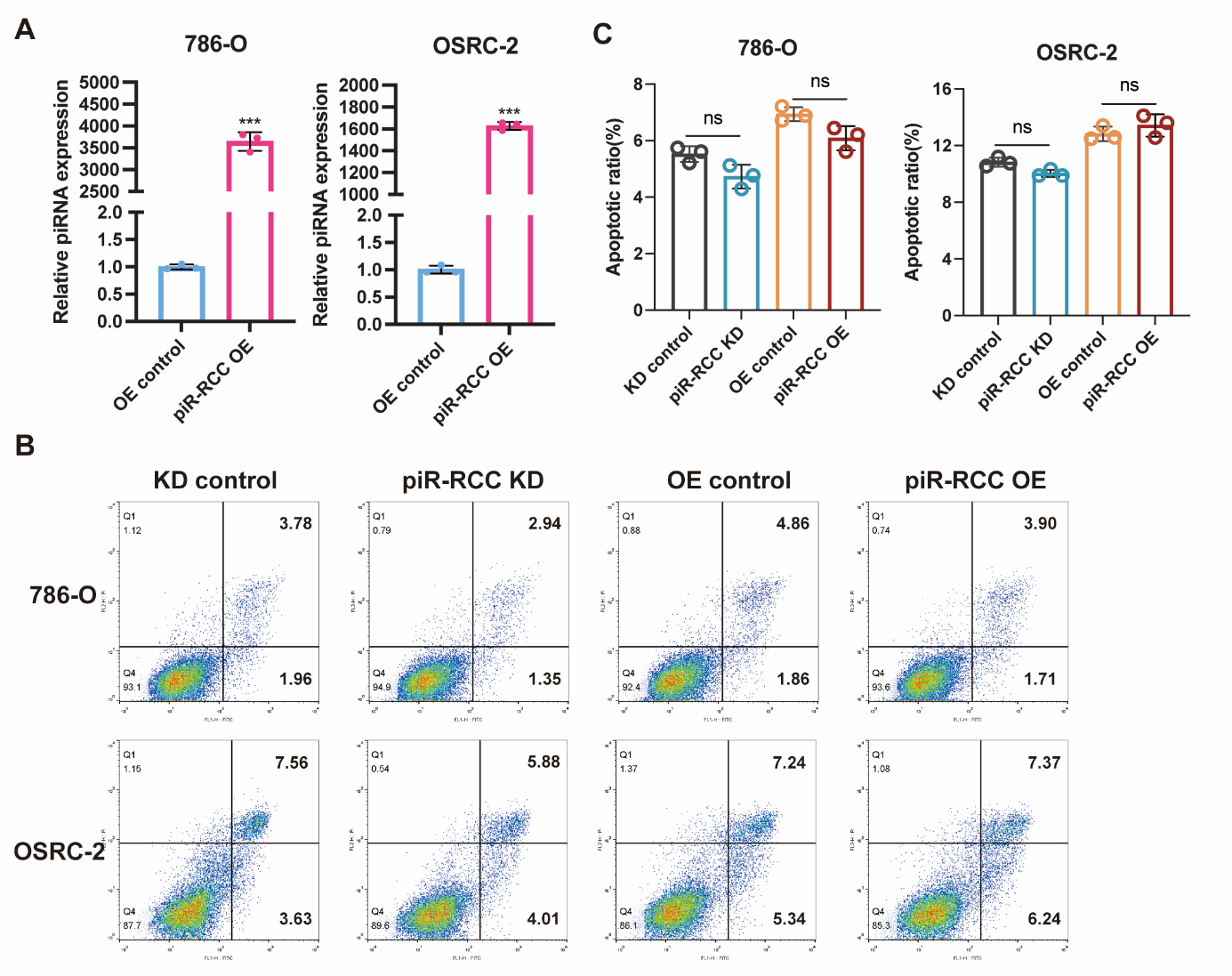


**Figure S2. piR-RCC has no significant effect on cell apoptosis.**

**(A)** The validation of the piR-RCC overexpression detected by qRT-PCR in 786-O and OSRC-2 cells. **(B, C)** Flow cytometric analysis of apoptotic ratio in 786-O and OSRC-2 cells. ***P < 0.001; ns, not significant. Data are presented as mean± SD; two-tailed unpaired t test was used for (A, C). ***P < 0.001; ns, not significant.


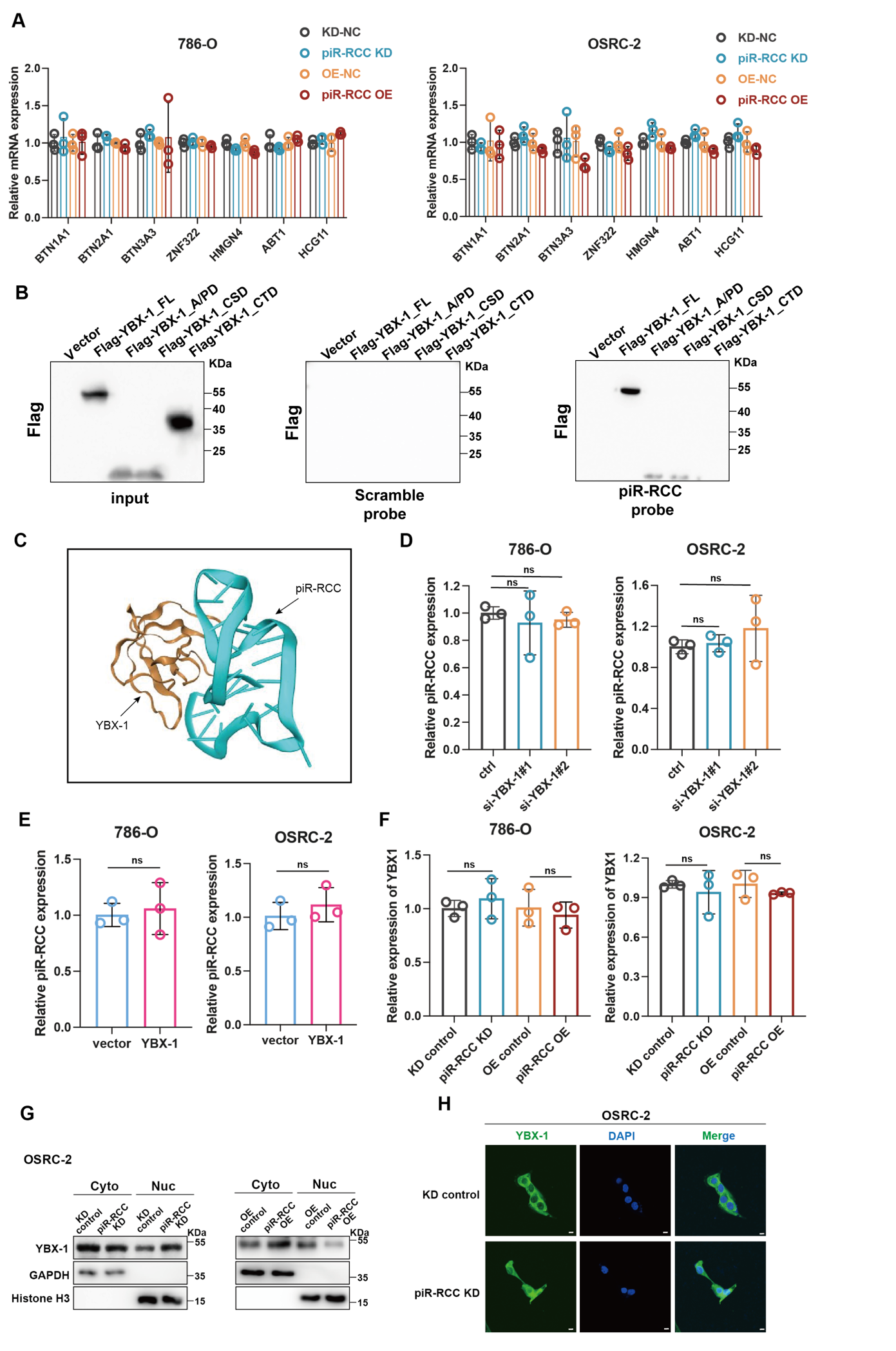


**Figure S3. piR-RCC interacts with YBX-1 proteins in RCC cells and suppresses its nuclear translocation**

**(A)** qRT-PCR was conducted to detect the expression of piR-RCC on nearby extrachromosomal genes in piR-RCC knockdown or overexpressing cells. **(B)** RNA pulldown assays showing the interaction between piR-RCC and YBX-1 truncations. **(C)** Molecular docking was conducted for the complex of YBX-1 with piR-RCC. Brown, YBX-1; Cyan, piR-RCC. **(D, E)** qRT-PCR analyssis of piR-RCC expression in YBX-1 knockdown or overexpression cells. **(F)** qRT-PCR analysis of YBX-1 mRNA expression in piR-RCC knockdown or overexpression cells. **(G)** WB analysis of YBX-1 in cytoplasmic and nuclear fractions of piR-RCC knockdown or overexpression OSRC-2 cells. **(H)** IF assays showed the subcellular localization of YBX-1 protein in piR-RCC knockdown OSRC-2 cells. Scale bar, 10μm. ns, not significant. Data are representative of three independent experiments and are shown as the mean ± SD; two-tailed unpaired t test was used for (A, D, E, F); ns, not significant.


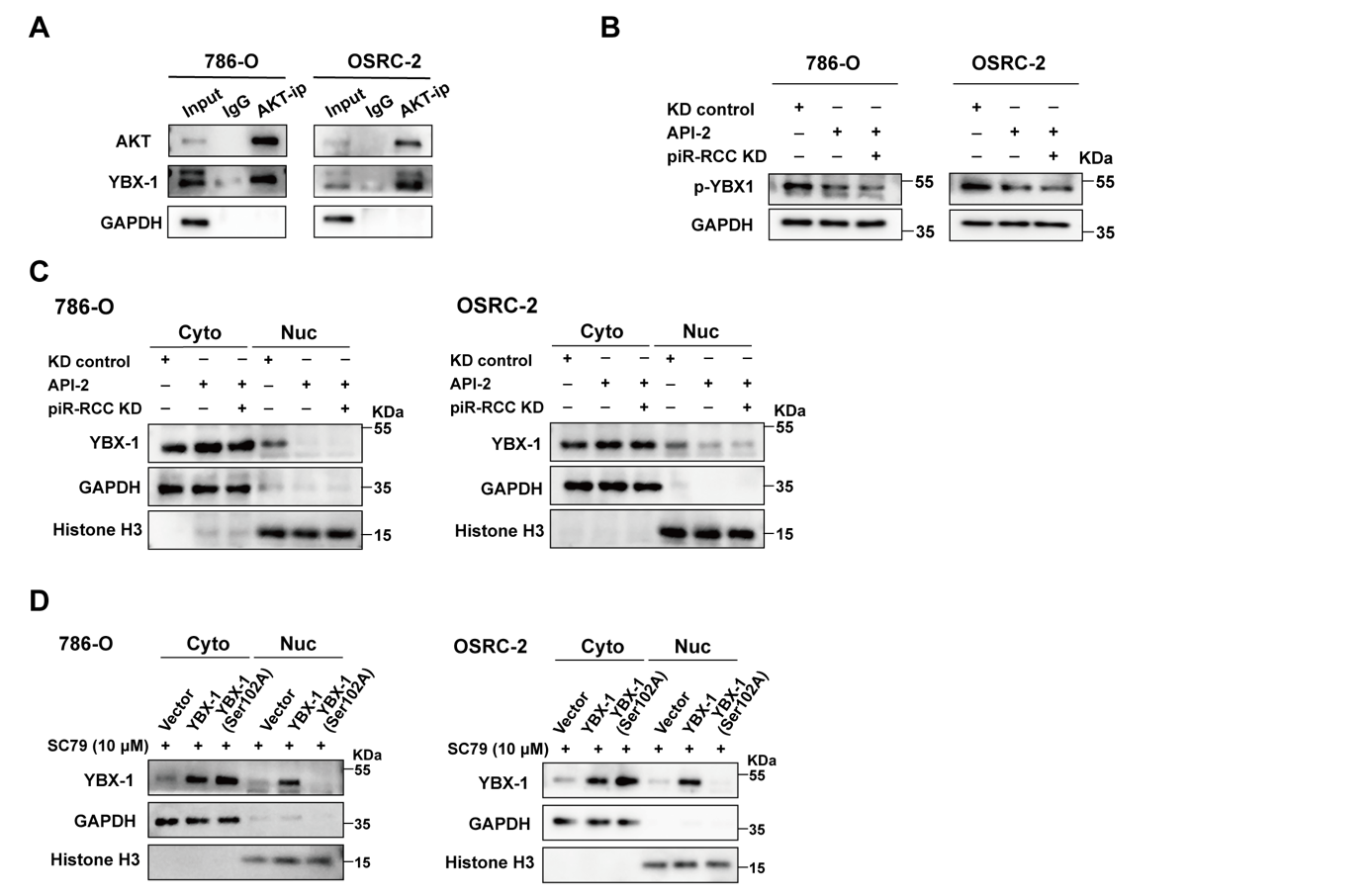


**Figure S4. YBX-1 S102 is a critical site for YBX-1 nuclear translocation.**

**(A)** Co-IP assays showing the interaction between AKT and YBX-1. **(B)** WB assay showing the p-YBX-1 expression with piR-RCC knockdown under API-2 treatment. **(C)** WB showing the cytoplasmic and nuclear fractions of YBX-1 in piR-RCC knockdown RCC cells under API-2 treatment. **(D)** WB assays showing that the cytoplasmic and nuclear fractions of YBX-1 in RCC cells transfected with YBX-1 wild type and YBX-1 S102A plasmids under SC79 treatment. Data are representative of three independent experiments.


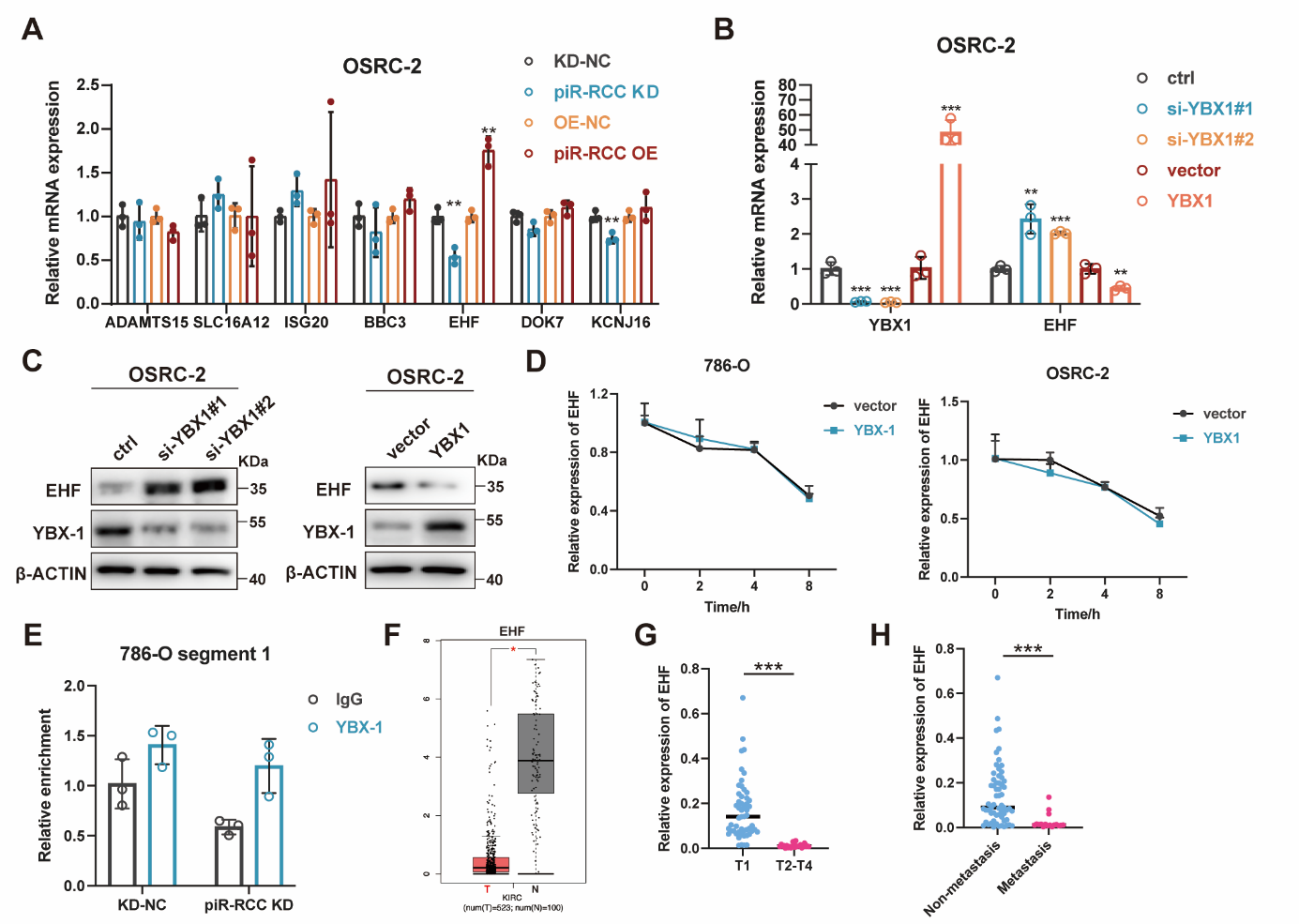


**Figure S5. piR-RCC and YBX-1 co-regulate EHF transcription.**

**(A)** qRT-PCR was conducted to detect the mRNA expression of candidate genes as indicated in OSRC-2 cells. **(B, C)** qRT-PCR and western blotting analysis of EHF expression in YBX1-knockdown or overexpressing OSRC-2 cells. **(D)** The RNA stability assays of EHF mRNA in RCC cells with YBX-1 overexpression. **(E)** YBX-1-CHIP-qPCR assays demonstrated that piR-RCC knockdown has a minimal effect on the binding efficiency between segment 1 and YBX-1. **(F)** The expression of EHF in Kidney renal cell carcinoma (KIRC); data were derived from GEPIA. **(G, H)** The expression of EHF in different stages and metastatic status within SRRSH cohort. Data are representative of three independent experiments and are shown as the mean ± SD; two-tailed unpaired t test was used for (A, B, G, H). **P < 0.01, ***P < 0.001; ns, not significant.


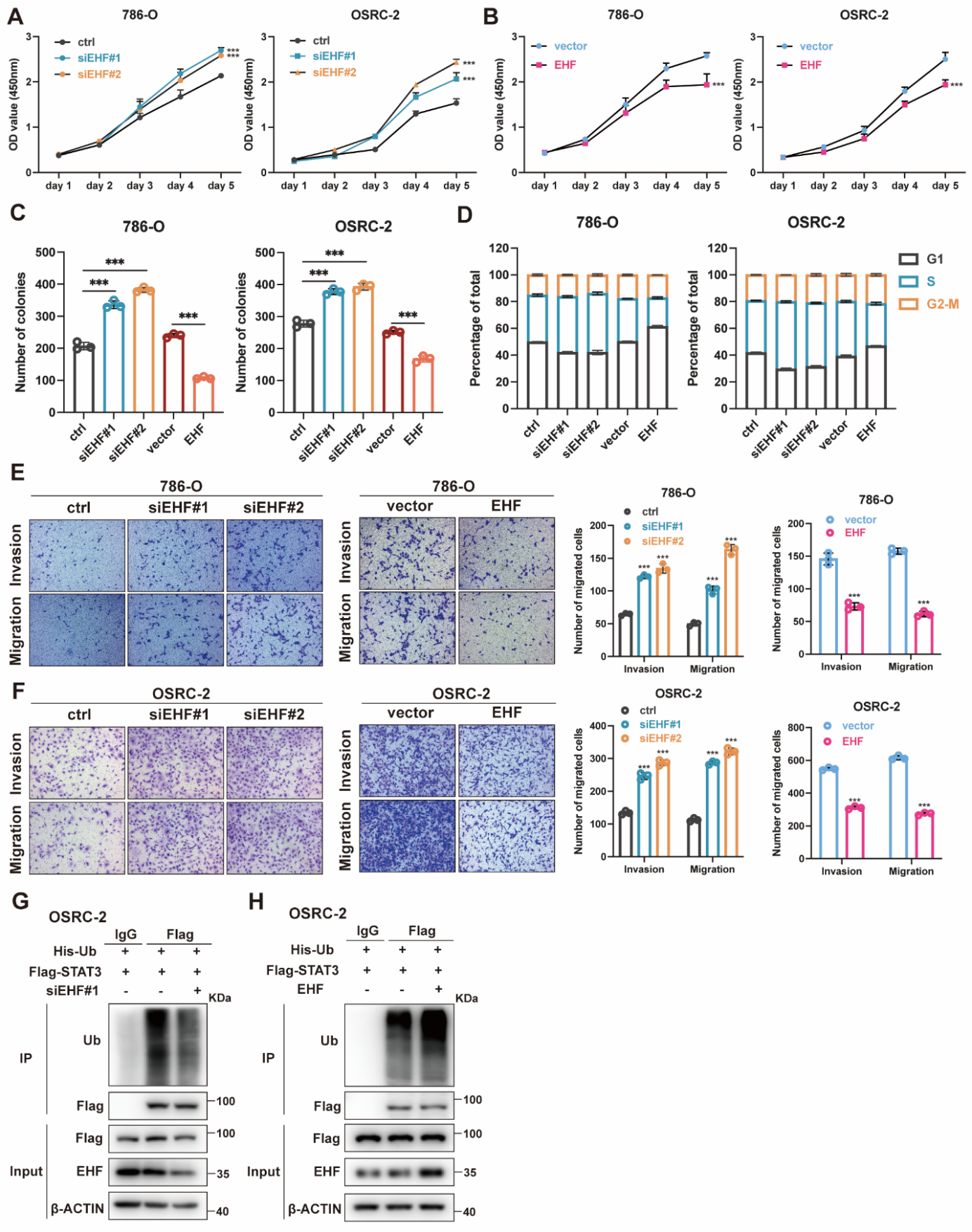


**Figure S6.** **EHF suppresses RCC cancer cells proliferation and migration**

**(A-C)** CCK-8(A, B) and colony formation (C) showing the proliferation ability of RCC cells with EHF knockdown or overexpression. **(D)** Flow cytometric analysis of cell cycle in EHF knockdown or overexpression cells. **(E, F)** Transwell assay to explore the effect of EHF on invasion and migration ability in indicated 786-O and OSRC-2 cells. **(G)** Western blotting assay was performed to detect the ubiquitination levels of STAT3 protein after EHF overexpression or knockdown in OSRC-2 cells. Data are representative of three independent experiments and are shown as the mean ± SD; two-tailed unpaired t test was used for (A-F). *P < 0.05, **P < 0.01, ***P < 0.001; ns, not significant.


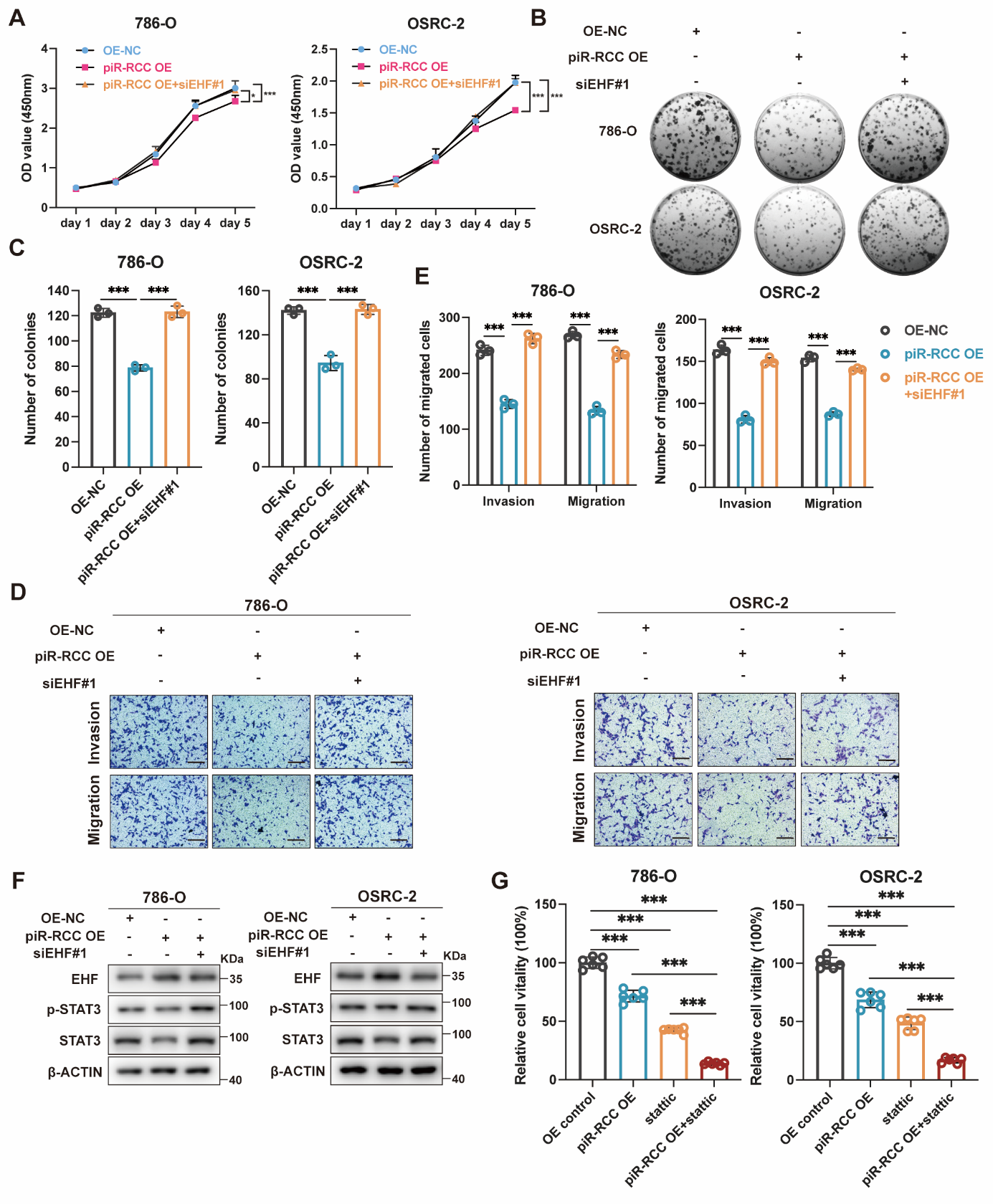


**Figure S7. piR-RCC impedes tumor progression via EHF-dependent mechanism**

**(A-C)** CCK-8(A) and colony formation (B, C) of RCC cells as indicated. **(D, E)** Representative images of transwell assay and its quantification data in piR-RCC overexpression cells transfected with or without EHF siRNA. **(F)** Western blotting of STAT3 and p-STAT3 protein expression upon piR-RCC overexpression with EHF knockdown or without. **(G)** Cell viability of control or piR-RCC overexpression cells treated with or without stattic. *P < 0.05, **P < 0.01, ***P < 0.001; ns, not significant. Data are representative of three independent experiments and are shown as the mean ± SD.


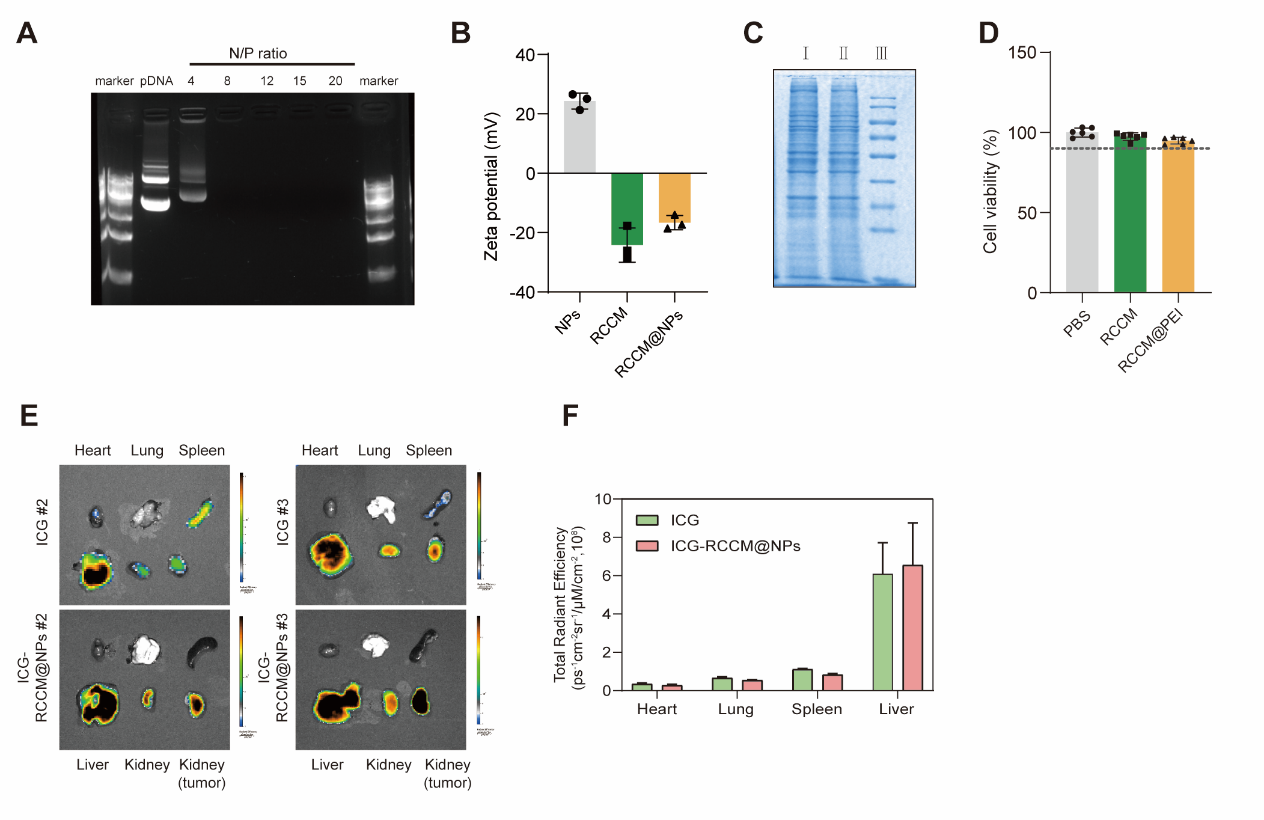


**Figure S8. Preparation, Characterization and biodistribution of RCCM@NPs**

**(A)** Agarose gel electrophoresis for PEI/pDNA with different N/P ratios (pDNA: plasmid DNA). **(B)** Zeta potential of NPs, RCCM, RCCM@NPs. **(C)** SDS-PAGE protein analysis stained with Coomassie Brilliant Blue as shown in. Ⅰ: RCC cell membrane; Ⅱ: RCCM@NPs; Ⅲ: marker. **(D)** CCK-8 assay of cell activity of different nanoparticles. **(E)** Ex vivo fluorescence images of ICG fluorescent dye accumulation in different organs 96 hours after intravenous injection. **(F)** Quantification of ICG intensity in different organs.

**Table S1**.

Clinical characteristics of RCC patients in SRRSH cohort

| Characteristics | Number (%) | piR-RCC expression | | P-value |
| --- | --- | --- | --- | --- |
|  |  | Low | High |  |
| Age (years) |  |  |  | 0.823 |
| <60 | 41 (51%) | 21 | 20 |  |
| ≥60 | 39 (49%) | 19 | 20 |  |
| Gender |  |  |  | 0.625 |
| Female | 24 (30%) | 11 | 13 |  |
| Male | 56 (70%) | 29 | 27 |  |
| TMN stage |  |  |  | 0.03 |
| I | 55 (69%) | 23 | 32 |  |
| II/III/IV | 25 (31%) | 17 | 8 |  |
| Metastasis stage |  |  |  | 0.014 |
| Non-metastasis | 63 (79%) | 27 | 36 |  |
| Metastasis | 17 (21%) | 13 | 4 |  |

**Table S2.**

Primers used for quantitative Real Time-PCR in this study.

| Gene Symbol | RT primer | Forward primer (5’→3’) | Reverse primer (5’→3’) |
| --- | --- | --- | --- |
| *piR-RCC* | *GTCGTATCCAGTGCGTGTCGTGGAGTCGGCAATTGCACTGGATACGACGGTCCTTC* | *CCCCCACTGCTAAATTTG* | *CAGTGCGTGTCGTGGAGT* |
| *piR-has-23588* | *GTCGTATCCAGTGCGTGTCGTGGAGTCGGCAATTGCACTGGATACGACTCTGGGTT* | *CGGAAGCGTGCTGGGCCCAT* | *CAGTGCGTGTCGTGGAGT* |
| *U6* | *AACGCTTCACGAATTTGCGT* | *CTCGCTTCGGCAGCACA* | *AACGCTTCACGAATTTGCGT* |

| Gene Symbol | Forward primer (5’→3’) | Reverse primer (5’→3’) |
| --- | --- | --- |
| *GAPDH* | *GTCTCCTCTGACTTCAACAGCG* | *ACCACCCTGTTGCTGTAGCCAA* |
| *YBX1* | *GGGGACAAGAAGGTCATCGC* | *CGAAGGTACTTCCTGGGGTTA* |
| *EHF* | *CCGCGACATCCTCTTGAACC* | *TTTACCCCATAGCTGAGCCAC* |
| *ADAMTS15* | *GACACTGCCATCCTCTTCACCA* | *CGTCCTCAATGACAGAGCAGCT* |
| *SLC16A12* | *GACCACCTCTTTGTCATCAGCG* | *ATCCACAGAGGAGGAATGCTGC* |
| *ISG20* | *ACACGTCCACTGACAGGCTGTT* | *ATCTTCCACCGAGCTGTGTCCA* |
| *BBC3* | *ACGACCTCAACGCACAGTACGA* | *CCTAATTGGGCTCCATCTCGGG* |
| *DOK7* | *GCCATCATGCTGGGCTTTGACA* | *AACTTGGTGCCTGGAGCCACTG* |
| *KCNJ16* | *GACAGTGAAGGGAGGATGACGA* | *TGGCTACTGCTTTGCGGTCAAG* |
| *BTN1A1* | *TGGATTCAGCTCCCTTTGACG* | *GAACCAGCGTAGCTCCAAGT* |
| *BTN2A1* | *AGGAGTACCGAGGAAGAACCA* | *TGTGATGTTGTGTATGACCAGG* |
| *BTN3A3* | *ATAAAGTGGAGCGACACCAAG* | *CAGCAACGAGATAGGCAGGG* |
| *ZNF322* | *GCACCAGGCAACTCACACT* | *CACACGCAGACCACTGATGT* |
| *HMGN4* | *GATCAGCTCGGTTGTCTGCTA* | *GCAGGGTTGTTCCCATCCTT* |
| *ABT1* | *ACAGACACTAGATGCGGAGGA* | *TACCTGGCACTACCCGTTTCT* |
| *HCG11* | *GTGACCACTCTGTCGCCATT* | *GTAGTACGGAGACCAACCGC* |
| *CHIP-Segment1* | *TCCTGGAAGGTTCAACAG* | *GCTTCACTGGGTCACAAA* |
| *CHIP-Segment2* | *CCGTAGATCCTCCCTAAT* | *GGTTTATTGACACTCCCTAT* |
| *CHIP-Segment3* | *CCCCTTCACCATCACTACAC* | *GACAAATCAGGCACAACCT* |

**Table S3**.

siRNAs used in this study.

| Gene Symbol | siRNA sequence (sense 5’→3’) |
| --- | --- |
| *si-YBX1#1* | *GGAUAUGGUUUCAUCAACATT* |
| *si-YBX1#2* | *CGUAACCAUUAUAGACGCUTT* |
| *si-EHF#1* | *GACGAAGACUGGUAUAUAATT* |
| *Si-EHF#2* | *GCCAGUGGCAUGAAAUUCATT* |

**Table S4**: Antibody used in this study

| Antibody | company | catalog number |
| --- | --- | --- |
| *YBX-1* | *Proteintech* | *20339-1-AP* |
| *GAPDH* | *Abcam* | *ab8245* |
| *AKT* | *CST* | *4685* |
| *p-AKT (ser473)* | *CST* | *4060* |
| *p-YBX-1(ser102)* | *CST* | *2900S* |
| *Histone H3* | *Abcam* | *ab1791* |
| *p-RSK* | *CST* | *9335S* |
| *p-ERK1/2* | *CST* | *4695* |
| *STAT3* | *CST* | *4904* |
| *p-STAT3* | *CST* | *9135L* |
| *β-actin* | *CST* | *3700* |
| *FLAG* | *Sigma* | *F1804* |
| *Ub* | *CST* | *58395S* |
| *GFP* | *Yoche* | *AYC30-100* |
| *ERK* | *CST* | *9102* |
| *RSK* | *CST* | *9355* |

**Table S5**.

Biotinylated probe and sequence FISH oligonucleotide probe

| probe | sequence |
| --- | --- |
| *piR-RCC probe* | *GGTCCTTCGAGCCGGATTCGAACCAGCGAC* |

**Table S6**.

Biotinylated probe and sequence

| probe | sequence |
| --- | --- |
| *scramble probe* | *5’ biotin-GAUACCAAGGACAUACGCUUAUGCAUGCUA* |
| *piR-RCC probe* | *5’ biotin-GGTCGCTGGTTCGAATCCGGCTCGAAGGACC* |

**Table S7**.

piR-RCC specific binding proteins based on MS

| TUBA1B |
| --- |
| MOGS |
| HEL32 |
| PDIA3 |
| PSMD2 |
| PKP1 |
| ACTR3 |
| KRT6B |
| PSMB1 |
| HEL-S-71p |
| DDX21 |
| MCM2 |
| ADAM10 |
| CPA4 |
| FLNB |
| BLMH |
| PTCD3 |
| FARSA |
| DMKN |
| TUBB |
| MSH2 |
| RPL11 |
| PRSS3 |
| RUVBL1 |
| MTIF2 |
| SEPTIN9 |
| QSOX1 |
| APMAP |
| KPNA3 |
| YOD1 |
| YWHAZ |
| PSMB6 |
| PFKL |
| KRT23 |
| POF1B |
| ARTS-1 |
| EXOC1 |
| UGT1A6 |
| PITRM1 |
| GDI1 |
| SEC62 |
| FGG |
| ATIC |
| PHB2 |
| RTN4 |
| EMC1 |
| HAL |
| CAPRIN1 |
| LBR |
| eIF3a |
| HNRNPDL |
| CCT3 |
| TPM1 |
| ITGB1 |
| HARS1 |
| PHB |
| ERP70 |
| PRSS2 |
| CCDC47 |
| A2ML1 |
| DHX15 |
| KRT13 |
| EDARADD |
| FLG |
| OLA1 |
| RPS9 |
| GAPVD1 |
| GSDMA |
| ANXA4 |
| CUL2 |
| DHX9 |
| ZC3H15 |
| SLURP2 |
| FGA |
| FGB |
| GLRX |
| NAP1L1 |
| CTSA |
| PLOD1 |
| FXR1 |
| AHCY |
| LUC7L3 |
| PCBP3 |
| PLBD1 |
| A2M |
| YBX1 |
| MICAL2 |
| CAPNS2 |
| TNPO2 |
| SERPINB13 |
| VPS35 |

**Grayscale value analysis of Western blot images in Figure 4.**


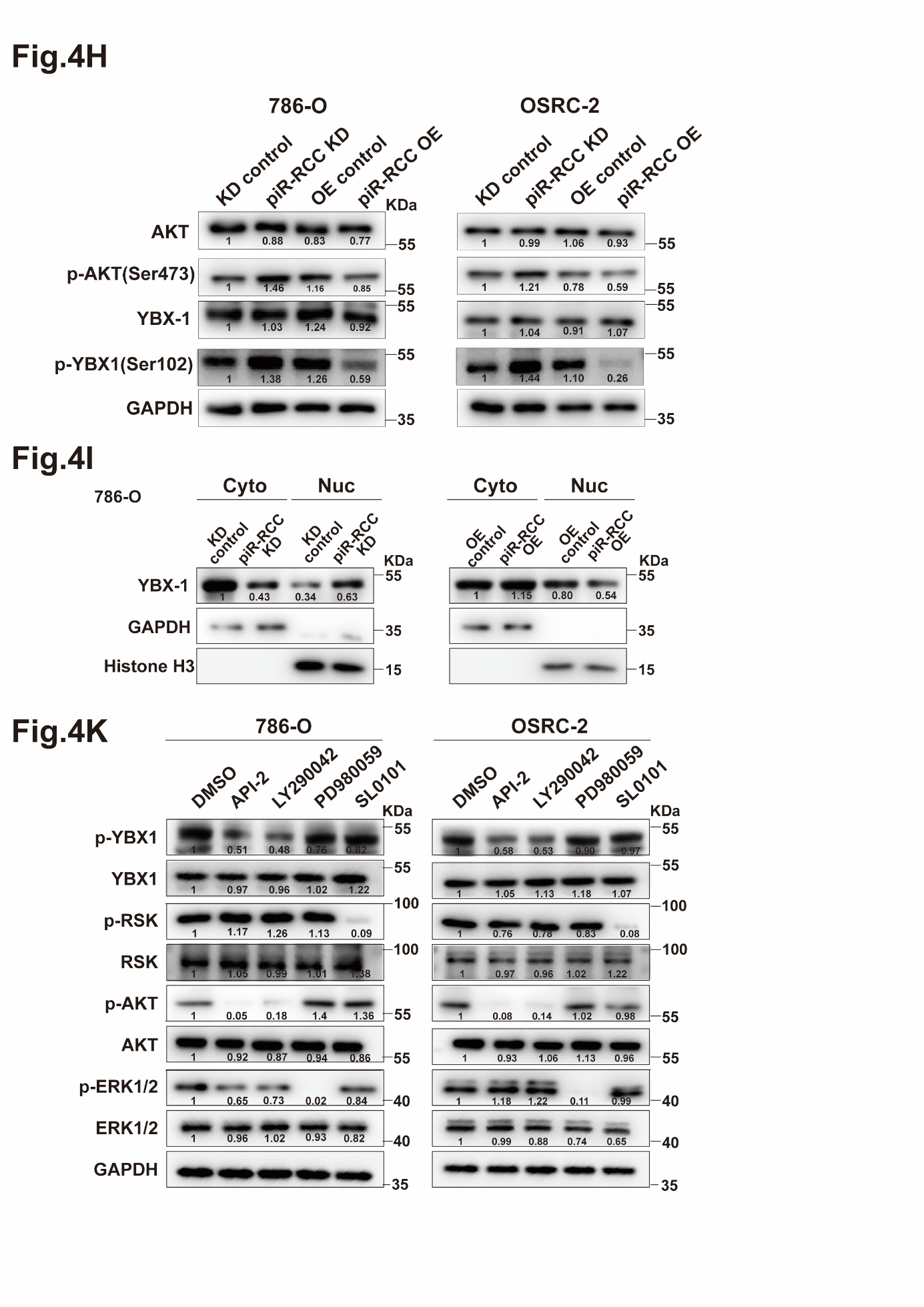


**Grayscale value analysis of Western blot images in Figure 5-6.**


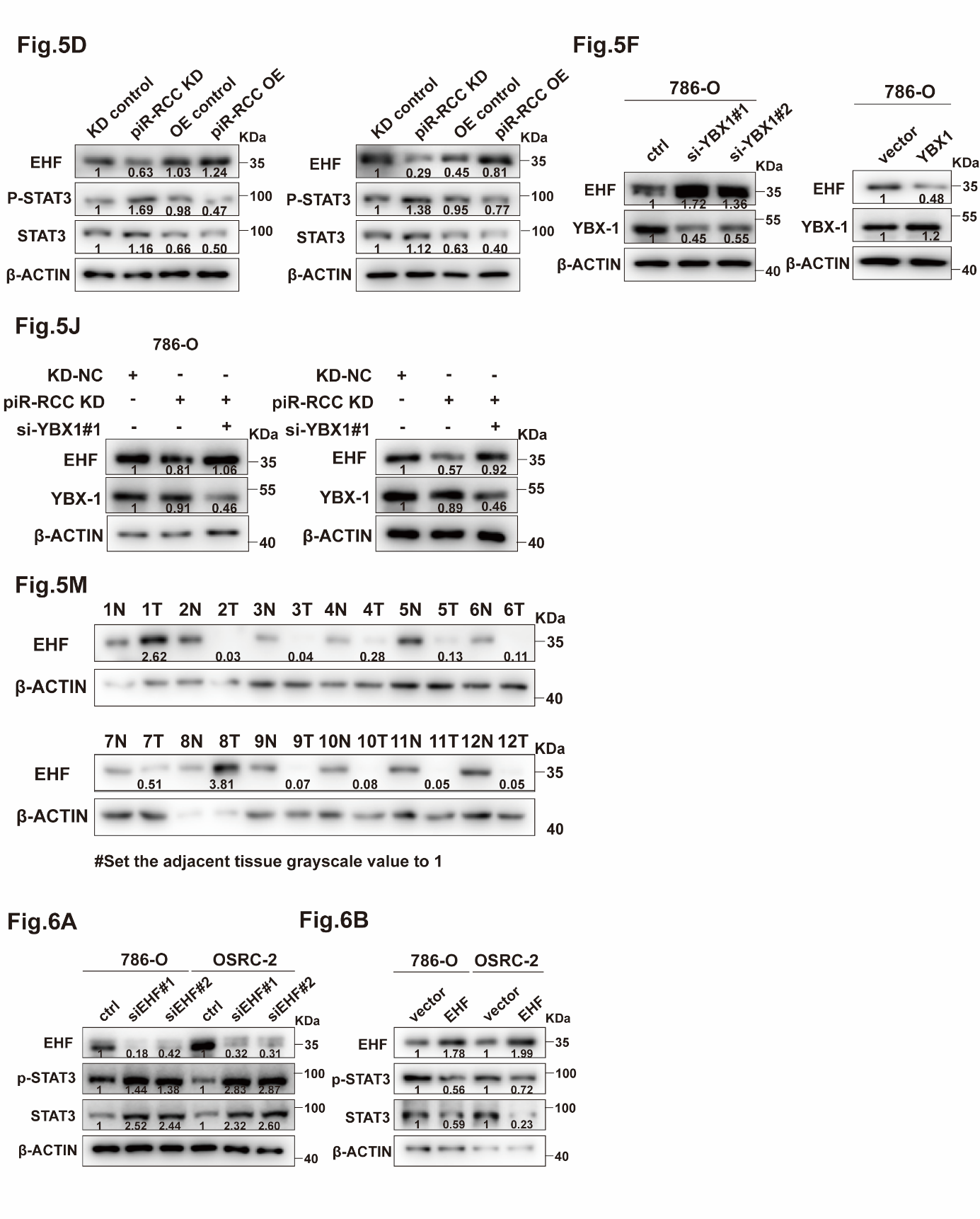


**Grayscale value analysis of Western blot images in Supplementary Materials**


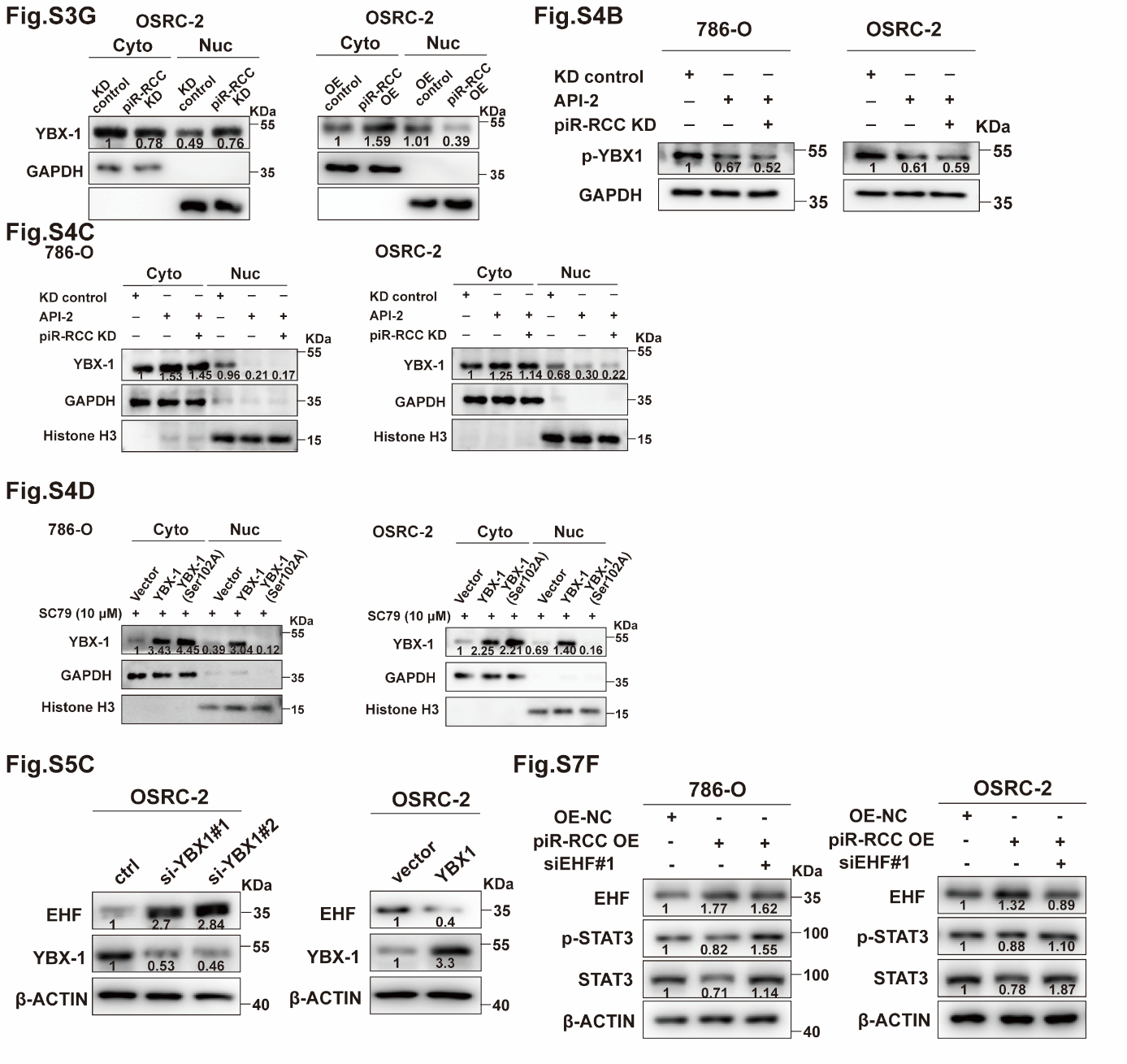

Supplement: Supplementary file 1 — Supporting Information [file ADVS-12-e14398-s001.docx]
